# An end-to-end method for predicting compound-protein interactions based on simplified homogeneous graph convolutional network and pre-trained language model

**DOI:** 10.1186/s13321-024-00862-9

**Published:** 2024-06-07

**Authors:** Yufang Zhang, Jiayi Li, Shenggeng Lin, Jianwei Zhao, Yi Xiong, Dong-Qing Wei

**Affiliations:** 1 School of Mathematical Sciences and SJTU-Yale Joint Center for Biostatistics and Data Science, Shanghai Jiao Tong University, Shanghai, 200240 China; 2 Peng Cheng Laboratory, Shenzhen, 518055 Guangdong China; 3 Zhongjing Research and Industrialization, Institute of Chinese Medicine, Zhongguancun Scientific Park, Meixi, Nanyang, 473006 Henan China; 4 State Key Laboratory of Microbial Metabolism, School of Life Sciences and Biotechnology, and Joint Laboratory of International Cooperation in Metabolic and Developmental Sciences, Ministry of Education, Shanghai JiaoTong University, Shanghai, China; 5 Shanghai Artificial Intelligence Laboratory, Shanghai, 200232 China

**Keywords:** Compound-protein interactions, Graph convolutional network, End-to-end learning, word2vec

## Abstract

Identification of interactions between chemical compounds and proteins is crucial for various applications, including drug discovery, target identification, network pharmacology, and elucidation of protein functions. Deep neural network-based approaches are becoming increasingly popular in efficiently identifying compound-protein interactions with high-throughput capabilities, narrowing down the scope of candidates for traditional labor-intensive, time-consuming and expensive experimental techniques. In this study, we proposed an end-to-end approach termed SPVec-SGCN-CPI, which utilized simplified graph convolutional network (SGCN) model with low-dimensional and continuous features generated from our previously developed model SPVec and graph topology information to predict compound-protein interactions. The SGCN technique, dividing the local neighborhood aggregation and nonlinearity layer-wise propagation steps, effectively aggregates K-order neighbor information while avoiding neighbor explosion and expediting training. The performance of the SPVec-SGCN-CPI method was assessed across three datasets and compared against four machine learning- and deep learning-based methods, as well as six state-of-the-art methods. Experimental results revealed that SPVec-SGCN-CPI outperformed all these competing methods, particularly excelling in unbalanced data scenarios. By propagating node features and topological information to the feature space, SPVec-SGCN-CPI effectively incorporates interactions between compounds and proteins, enabling the fusion of heterogeneity. Furthermore, our method scored all unlabeled data in ChEMBL, confirming the top five ranked compound-protein interactions through molecular docking and existing evidence. These findings suggest that our model can reliably uncover compound-protein interactions within unlabeled compound-protein pairs, carrying substantial implications for drug re-profiling and discovery. In summary, SPVec-SGCN demonstrates its efficacy in accurately predicting compound-protein interactions, showcasing potential to enhance target identification and streamline drug discovery processes.

**Scientific contributions**

The methodology presented in this work not only enables the comparatively accurate prediction of compound-protein interactions but also, for the first time, take sample imbalance which is very common in real world and computation efficiency into consideration simultaneously, accelerating the target identification and drug discovery process.

**Supplementary Information:**

The online version contains supplementary material available at 10.1186/s13321-024-00862-9.

## Introduction

Identification of interactions between compounds and proteins holds immense importance in various realms. Specifically, discovering new drugs is globally significant, both in academic research and commercial endeavors. The exploration of compound-protein interactions (CPIs) is pivotal in identifying compounds that interact with specific molecular targets. This process is fundamental for various purposes such as drug discovery, target identification, network pharmacology, comprehending protein functionalities, and more [1, 2]. However, the task of identifying new compounds along with their corresponding protein targets remains a formidable challenge, primarily due to the limited comprehension of the intricate relationships between the chemical space and proteomic space. Wet experimental tests are crucial methods utilized to assess the safety and effectiveness of novel drugs or treatment strategies. Nonetheless, these methods are often proven to be costly and time-consuming, demanding substantial resources. The evaluation process involves in vivo testing, which examines the effects of a drug or treatment within a living organism. However, this can be intricate and problematic due to ethical concerns and the inherent variability of biological systems. Alternatively, in vitro testing investigates the effects of drugs or treatments in a controlled laboratory setting outside a living organism, which also tends to be time-consuming and expensive due to the requirement for specialized equipment and expertise [3].

Recently, the use of machine learning (ML) and deep learning (DL) algorithms like Random Forest (RF) [4], Support Vector Machine (SVM) [5], Deep Neural Network (DNN) [6], Gradient Boosting Decision Tree (GBDT) [7] has speeded up the CPI identification process by enabling the development of novel compounds candidates with enhanced efficiency, efficacy, and quality [8]. Chen et al. [9] proposed TransformerCPI to improve compound-protein interaction prediction by sequence-based deep learning with self-attention mechanism. Li et al. [10] developed MONN, a multi-objective neural network capable of accurately predicting binding affinities between compounds and proteins. Additionally, MONN effectively captures the non-covalent interactions between compounds and proteins. DEEPScreen [11] utilized convolutional neural networks with 2-D structural compound representations. These techniques have been used to discover targets that are more specific and effective, and to identify novel compounds that can be further optimized for therapeutic use. By leveraging large datasets and computational models, ML and DL algorithms can predict the interactions between compounds and their targets, analyze the pharmacological properties of candidate compounds, and optimize the chemical structures of molecules to improve their potency, selectivity, and safety profiles. Moreover, these techniques can significantly shorten time and costs associated with traditional drug discovery methods, which rely on trial-and-error experiments and animal testing. Overall, the integration of ML and DL algorithms into CPIs prediction holds great promise for the development of safer, more effective, and more affordable treatments for a wide range of diseases. However, fewer methods based on ML and DL for predicting CPIs use end-to-end representation learning. Instead, they relied on hand-extracted and well-designed compounds and protein features as input to the neural network. Using molecular fingerprints and protein structures as input features requires some prior knowledge about the data and involves hand-crafted features. While end-to-end learning has proven to be an effective method for feature representation, it is not commonly used in biological problems. In the case of the CPI problem, compounds or proteins can be represented as sequences where each character represents an atom or amino acid which were similar with ‘sentences’ in the natural language processing. Therefore, there are a lot of potentials for considering end-to-end learning of CPI feature representations based on these assumptions. Various protein language models and compound language models have been proposed for feature representation. Examples include ProtVec [12] and SMILES2Vec [13] based on word2vec [14] methodology model, ProteinBERT [15] and Knowledge-based BERT [16] for compounds, as well as large language models like ESM-1b [17].

Graph convolutional networks (GCN) [18] have achieved significant advancement in processing network or data with graph-structure and are deemed a promising solution to the CPIs problems. GCN plays a vital role in investigating intricate biological systems, which are represented as graphs made up of nodes (i.e., biomolecules) and edges (i.e., connections between biomolecules), such as CPIs analyzed in this study. Previous studies demonstrated that biomolecules, such as small molecules and proteins, carry out their functions not only individually but also through interactions with other biomolecules. As a result, network topology should be considered to predict interactions among biomolecules. To date, GCN has been extensively applied in numerous real-world tasks, yielding satisfactory results in drug-target interaction or affinity prediction [19–22], drug-drug interaction prediction [23, 24], disease-gene association recognition [25–27], and so on. In biological tasks like CPIs, the number of pertinent entities (e.g., genes, compounds, proteins, etc.) is typically enormous. The most typical hurdle is the “neighbor explosion” phenomenon encountered when dealing with complex large graphs (the complexity of node representation and stochastic gradient calculation will exponentially increase with the increasing number of message passing layers), and the over-smoothing or overfitting issues caused by stacking multiple layers of GCN (as the neural network goes deeper, nodes tend to have similar representations after aggregation operations). Researchers have proposed various graph sampling techniques to reduce the number of nodes involved in message passing, thereby lowering training costs. The most common techniques include node sampling (such as GraphSAGE [28], PinSage [29], VRGCN [30]), layer sampling (such as FastGCN [31], ASGCN [32]) and edge sampling [28]. In addition to training complexity issues, there are still challenges on accuracy and scalability [33, 34].

In real-world scenarios, the number of observed CPIs is often significantly lower than the potential interactions that could exist. Therefore, using unbalanced datasets more accurately reflects the natural distribution of positive and negative samples in CPI prediction tasks. However, many existing methods for CPI prediction are trained and evaluated using balanced datasets, as observed in [35–37]. In balanced datasets, models might achieve artificially high accuracy due to being prone to predicting the majority class. This can be misleading and does not provide an accurate assessment of a model’s performance. Therefore, it becomes imperative to address the challenge posed by natural imbalanced data, even though training models on imbalanced datasets remain a significant hurdle for machine learning techniques [38]. Unbalanced datasets force models to learn the underlying patterns of interactions, leading to more reliable evaluations. Imbalanced datasets can improve a model's sensitivity to true positive predictions. Sensitivity is crucial in CPI prediction, as accurately identifying existing CPIs is essential for drug development and repurposing. Successfully predicting interactions in an imbalanced setting indicates better generalization and robustness of the model when applied to real-world situations.

**Fig. 1:**
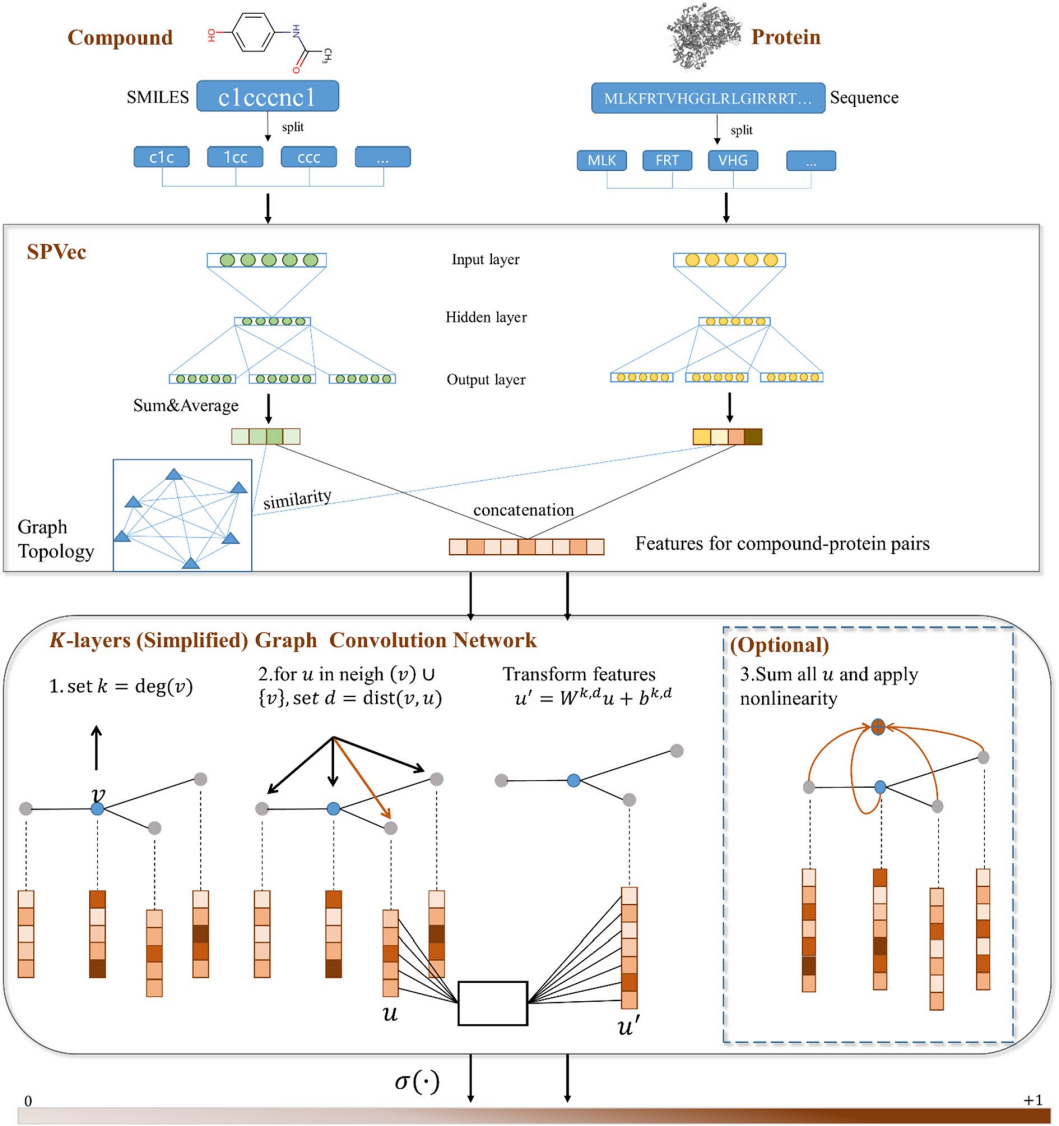
The whole pipeline for CPI prediction. There are three steps for CPI prediction task: (i) feature representation via SPVec method, (ii) graph construction based on feature concatenation and feature similarity and (iii) simplified GCN model with $\text{K}$-layers

In this study, we proposed an end-to-end approach called SPVec-SGCN-CPI utilizing a simplified homogenous GCN model by concatenating compounds and protein features derived from the SPVec [39] model. Figure 1 illustrated the whole pipeline for CPI prediction. There are three steps for CPI prediction task: (i) feature representation via SPVec method, (ii) graph construction based on feature concatenation and feature similarity and (iii) simplified GCN model with $\text{K}$-layers. The SGCN technique separates the local neighborhood aggregation and nonlinearity layer-wise propagation steps, effectively aggregating $K$-order neighbor information while preventing neighbor explosion and speeding up training [40]. This makes the training process more efficient and allows the algorithm to handle larger graphs. The SPVec-SGCN-CPI method's performance was evaluated across three datasets, comparing it against four ML- and DL-based methods and four state-of-the-art methods. Experimental results demonstrated that SPVec-SGCN-CPI outperformed ML, DL and state-of-art methods, particularly excelling in unbalanced datasets. In sum, SPVec-SGCN demonstrates its capacity in reliably predicting CPIs, exhibiting potential to enhance target identification and streamline drug discovery processes.

## Method

### Datasets

**Fig. 2:**
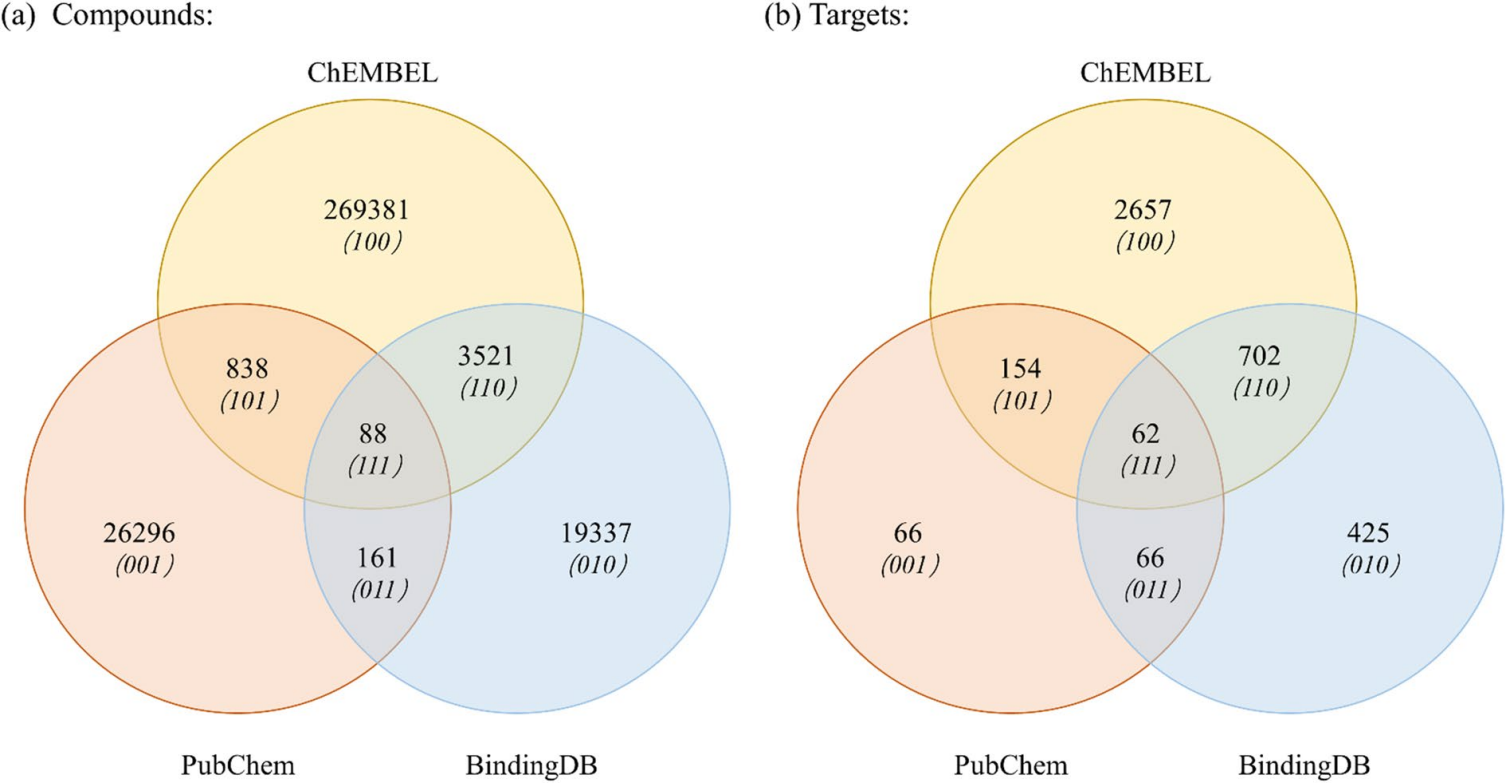
Numbers of compounds (**a**) and protein targets (**b**) that are unique or common in the ChEMBL, BindingDB and PubChem datasets. Numbers in parentheses indicate the inclusion relationship of different groups, 1 means containment, 0 means non-containment, and position indicates the group in which it is located

ChEMBL [41], BindingDB [42] and PubChem [43] are commonly used and frequently reported databases of chemical molecules and their biological activities. Affinity data for protein–ligand complexes are curated from published literature in major medicinal chemistry journals, and the data have been manually annotated to ensure the reliability. Supplementary Table S1 shows the numbers of compounds, target and their interactions obtained from three data source mentioned above as of October 2023. While many types of assays (IC50, AC50, EC50, ${K}_{i}$, ${K}_{d}$) has been widely used to quantify the potency of compounds in inhibiting the activity of a biological target, IC50 is most commonly reported in experimental studies because determination process of ${K}_{i}$/${K}_{d}$ is slightly cumbersome. Either a low IC50 value or a low Ki/Kd value indicates high binding affinity [44]. In order to maximize the utilization of our collected data, we selected IC50 as the primary quantitative measure. We firstly got rid of compound-protein pairs that had missing IC50 values and inorganic compounds because of low drugability. By following the activity threshold discussion in [45, 46], compound-protein pairs with IC50 values < 100 nM were selected as positive samples and compound-protein pairs with IC50 values > 10000 nM were selected as negative samples. It is worth noting that this threshold is variable. It can adjust the IC50 value [47–49] or classify positive and negative samples based on ${K}_{i}$ or ${K}_{d}$ values [50–53]. Table S2 lists the different criteria adopted by researchers, with related analyses following Table S2. According to data source, we used ChEMBL dataset as training data and the other two datasets as test data. Table 1 shows the final numbers of entries in three datasets obtained from ChEMBL, BindingDB and PubChem, respectively. Unlike the ChEMBL and BindingDB datasets, where the positive and negative sample quantities are relatively close, the ratio of positive to negative samples in the PubChem dataset is approximately 1:81, indicating a highly imbalanced distribution. This is in line with real-world phenomena because most compound-protein pairs are unmarked data or negative samples. The PubChem dataset addresses the sample imbalance issue, which many other studies have not considered. Figure 2 shows the numbers of compounds (Fig. 2a) and protein targets (Fig. 2b) that are unique or common in the ChEMBL, BindingDB and PubChem datasets. Each CPI entry in the three datasets is unique with  no overlap. It can be observed that the overlap of samples (compounds or targets) among the three datasets is very low. BindingDB and PubChem are suitable for use as test sets.

**Table 1:** Numbers of entries in three datasets obtained from ChEMBL, BindingDB and PubChem, respectively

	Datasets	Compounds	Targets	Positive samples	Negative samples	Total samples
Training set	ChEMBL	273652	3451	256590	169642	426232
Test sets	BindingDB	33916	1131	14265	14191	28456
	PubChem	27307	224	449	36581	37030

### Feature representations

In our previous study, we proposed SPVec [39] method to learn feature representation of small compounds (SMILES format) and target proteins sequences. SPVec, which was inspired by word2vec, uses the Skip-gram [54] model. The Skip-gram model is a type of neural network architecture that is used to predict the context words surrounding a target word in a sentence or text. The basic idea is to learn a set of distributed representations (vectors) for each word in the vocabulary, such that similar words have similar vectors. The Skip-gram model is trained on a large corpus of text data, and it learns to predict the probability of observing a context word given a target word. One of the advantages of using the skip-gram method is its ability to capture the semantic relationships between words. Negative-sampling method is used to train SPVec model, which helps to reduce computational complexity and to enhance simultaneously the quality of word vectors and to accelerate the training speed. Given a subset $NEG\left(w\right)( NEG\left(w\right)\ne \varnothing$) and $\forall \widetilde{w}\in D$, the probability of word vector is

1 $$p\left( {u\left| {\tilde{w}} \right.} \right) = \left[ {\sigma \left( {V\left( {\tilde{w}} \right)^{T} \theta^{u} } \right)} \right]^{{L^{{\text{w}}} \left( u \right)}} ,\left[ {1 - \sigma \left( {V\left( {\tilde{w}} \right)^{T} \theta^{u} } \right)} \right]^{{1 - L^{{\text{w}}} \left( u \right)}}$$

where ${L}^{w}(\widetilde{w})$ is the label of word$w$, $\sigma (\bullet )$ is sigmoid function, $\theta$ are parameters of latent word vectors. The following function is maximized for sample$(w,Context\left(w\right) )$:

2 $$g\left( {\text{w}} \right) = \mathop \prod \limits_{{\tilde{w} \in Context\left( {\text{w}} \right)}} \,\mathop \prod \limits_{{{\text{w}} \in \left\{ u \right\} \cup NEG^{{\tilde{w}}} \left( {\text{w}} \right)}} p\left( {u{|}\tilde{w}} \right){ }$$

where ${NEG}^{\widetilde{w}}(w)$ denotes as a subset generated from negative samples during processing words $\widetilde{w}$. The following objective loss function $\mathcal{L}$ is maximized by the stochastic gradient descent (SGD) method:

3 $${\mathcal{L}} = \log G = \log \mathop \prod \limits_{{{\text{w}} \in {\mathcal{C}}}} g\left( {\text{w}} \right)$$

SPVec learned distributed representations (vectors) for each category instead of one-hot encoding. To represent drug molecules, we treated SMILES as “sentences” and each atom as a “word”. For protein sequences, we regarded them as “sentences” and every three amino acids that not overlap to each other as a “word”. Since SMILES strings have different representations for the same chemical structure, we converted the original SMILES in datasets to canonical SMILES to ensure the consistency and quality of the generated features. Our previous work has demonstrated that SPVec is superior to the features of manual design and SPVec can avoid the sparseness problem and reduce the number of bit collisions. And we discuss the performance of different word vector dimensions and context window size for word vectors. However, previously, the influence of different corpus on word2vec was ignored. Here, we designed three corpuses to explore the sensitivity of word2vec to corpus quality: (1) Corpus_1 only contains ~ 273 K compounds and 3451 proteins in training phase; (2) Corpus_2 contains ~ 335 K compounds and 4806 proteins in all three datasets (i.e. ChEMBL, BindingDB and PubChem); (3) Corpus_3 contains ~ 2.4 M compounds and ~ 15 K proteins in ChEMBL. That is, SPVec was pre-trained by external data. We proposed two methods to explicitly address the limitations associated with word2vec. Handling Out-Of-Vocabulary (OOV) words is a significant obstacle for small corpus size (corpus_1). One common solution is assigning random vectors to OOV words. Here we replace random vectors with averaged vectors of ‘words’ (i.e. compounds and protein segments). Large corpus (corpus_3) may contain noisy data, we filtered molecules with Tanimoto similarity > 80% and proteins with sequence similarity > 80%. The improved corpus based on methods mentioned above are named as corpus_1_imp and corpus_3_imp, respectively. Besides, to make sure no data about new protein or a new SMILES or both was leaked in pre-trained and training process, Corpus_3_only were constructed by removing proteins and compounds in testsets (i.e. BindingDB and PubChem).

According to Tomas [55], improving the range can enhance the quality of word vectors, but it comes at the cost of increased computational complexity. The training complexity of Skip-gram model can be expressed as:

4 $$Q = C \times \left( {D + D \times log_{2} \left( V \right)} \right)$$

Here, *C* represents the maximum distance between words, $\text{V}$ represents real value vector $V\left(w\right)$ for any word in dictionary $D$.

### Simplified graph convolutional networks

GCN is one type of neural network specifically developed to handle data with graph structure. In convolutional neural networks (CNN), convolution operation works by sliding a filter over the input image and applying a dot product between the filter and the input at each location [56]. This operation can be extended to graph data by defining a filter as a weight matrix that is shared across all nodes in the graph. The output of the convolution operation is then calculated by taking the dot product between the weight matrix and a node feature matrix, where the node feature matrix contains feature vectors for all nodes in the graph.

A particular graph-based neural network model is theoretically motivated by the layer-wise back propagation rule below. Define

5 $${\text{S}} = \,\tilde{D}^{{ - \frac{1}{2}}} \tilde{A}\tilde{D}^{{ - \frac{1}{2}}}$$

Here, $\widetilde{\text{A}}=A+{I}_{N}$, where ${I}_{N}$ is the identity matrix and $A$ is adjacency matrix of graph *G*. $\widetilde{\text{D}}$ is the degree matrix of $\widetilde{\text{A}}$.

The representation updating rule of the $k$-th layer is:

6 $${\text{H}}^{\left( k \right)} \leftarrow {\text{ReLU}}\left( {SH^{{\left( {k - 1} \right)}} \Theta^{\left( k \right)} } \right)$$

The weight matrix ${\Theta }^{\left(k\right)}$ is specific and trainable to each layer. ${\text{H}}^{\left(k\right)}\in {\mathbb{R}}^{N\times D}$ is the matrix of activations in the *h*^th^ layer.

For binary classification, the predicted class $\widehat{\mathbf{Y}}$ in a $k$-layer GCN can be expressed as:

7 $$\hat{Y}_{{{\text{GCN}}}} = sigmoid\left( {SH^{{\left( {k - 1} \right)}} \Theta^{\left( k \right)} } \right)$$

where $sigmoid(x)=\frac{1}{1+{e}^{-x}}$ acts as a normalizer among two classes.

For traditional multilayer perceptron (MLP), greater depth enhances expressivity by enabling the formation of feature hierarchies. For instance, features in the next layer build upon those of the first layer. Feature propagation is the key factor that sets a GCN apart from a MLP. In GCNs, layers serve an additional crucial role: at each layer, hidden node representations are obtained by average among neighbors situated one hop away. Consequently, after $k$ layers, a node incorporates feature information from all nodes located $k$-hops away in the graph. This effect resembles CNN, where depth expands the receptive field of inner node features [57]. While convolutional networks significantly benefit from increased depth [58], MLPs typically derive little advantage beyond 4 or 5 layers.

**Fig. 3:**
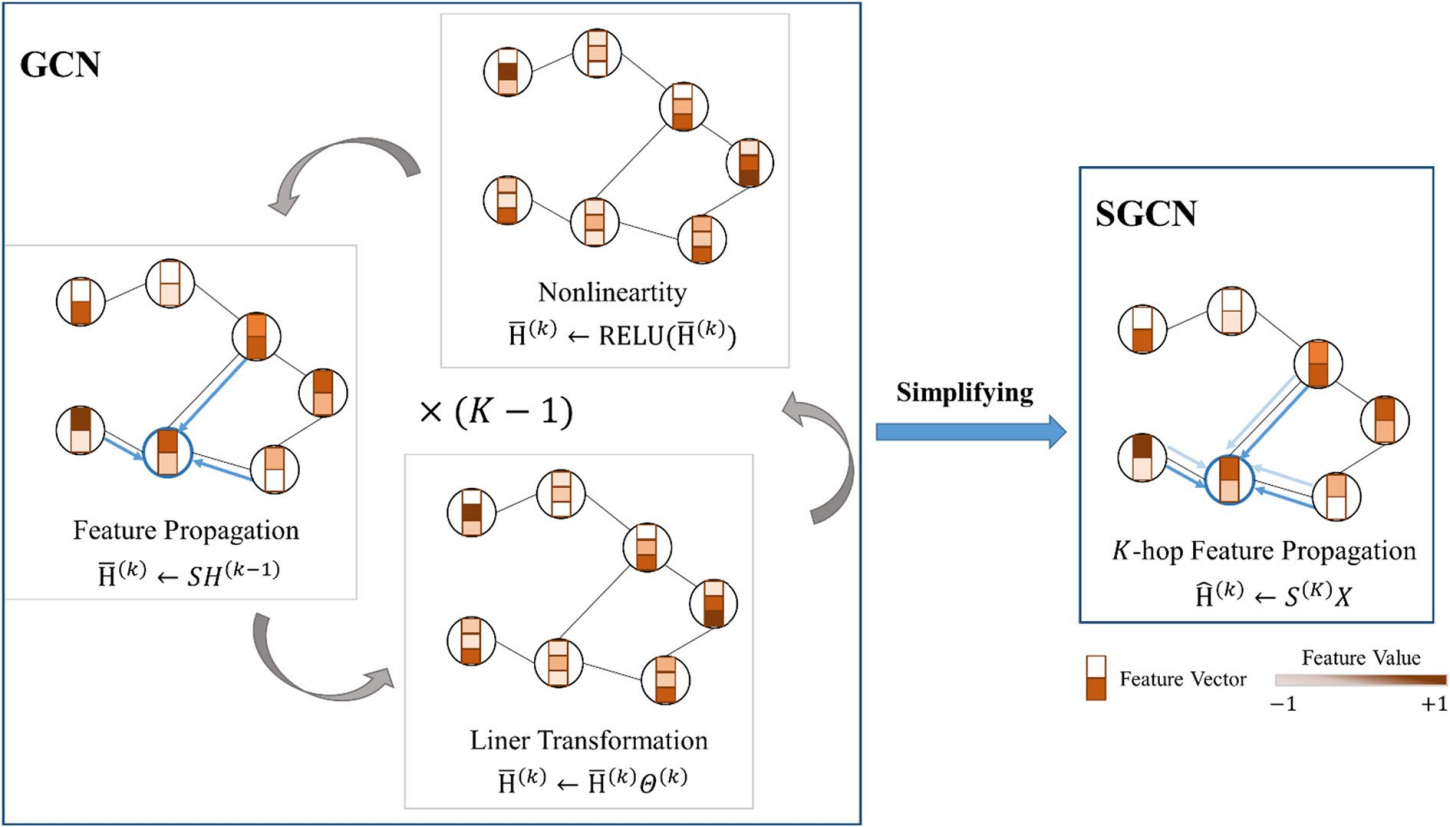
Schematic layout comparison between GCN and SGCN. In the left, the GCN iteratively transforms feature vectors across $K$ layers before employing a linear classifier on the ultimate representation. In contrast, the right showcases the SGCN, which simplifies the entire process to a single step of $K$-hop feature propagation

Our hypothesis suggests that GCN performs well on graph data for two key reasons. (1) local neighborhood aggregation: GCN can effectively capture the local neighborhood information of each node. By using the features of a node and its immediate neighbors for convolution, GCN aggregates information from surrounding nodes, incorporating their information into the representation of each node. (2) nonlinearity layer-wise propagation: GCN models typically employ nonlinearity layer-wise propagation, where each layer depends on the output of the previous layer. This layer-wise propagation effectively preserves and propagates information through the layers, allowing the model to gradually capture more extensive graph structural information and complex nonlinear relationships in the input data. These two parts can be executed separately. Figure 3 shows a schematic layout comparison between GCN and Simplified GCN (SGCN). SGCN eliminates the nonlinear transition functions in each layer, retaining only the final sigmoid to generate probabilistic outputs in a range of 0–1. The resultant model is linear, yet maintains the same increased receptive field characteristic of a $k$-layer GCN and can be freely combined with nonlinearity layer-wise propagation.

The predicted class $\widehat{\text{Y}}$ in a $k$-layer SGCN can be written as:

8 $$\hat{Y}_{{{\text{SGCN}}}} = sigmoid\left( {{\text{S}}^{k} {\text{X}}\Theta } \right)$$

Equation 8 yields a straightforward and intuitive understanding of SGCN. By delineating between feature representation and classification, SGCN comprises a fixed and parameter-free feature smoothing component $\widehat{\text{X}}={\text{S}}^{k}\text{X}$, succeeded by a linear logistic regression classifier $\widehat{\text{Y}}=\mathit{sigmoid}\left(\widehat{\text{X}}\Theta \right)$. As the computation of $\widehat{\text{X}}$ involves no weights $\Theta$, it is basically equivalent to feature transformation. Consequently, the entire model training process simplifies to binary logistic regression on the transformed features $\widehat{\text{X}}$. SGCN inherently scales well to very large graph sizes, making the training of SGCN significantly faster compared to GCNs. For a $n$-layer GCN, we use 1 or 2 layer nonlinearity propagation to capture complex nonlinear relationships and the other can directly execute $k$-layer local neighborhood aggregation by SGCN.

To construct the graph we need for our task, we use compound-protein pairs as nodes. The nodes have 200 dimensions of features obtained using SPVec. The adjacency matrix is obtained from the similarity among vectors of nodes representation. The label of each node indicates whether there is an interaction between the compounds and proteins. Table S3 showed detailed information about hyperparameters and architectures of SGCN model.

### Model evaluation

The evaluation process serves as an important step in determining the overall effectiveness of the model and ensuring its future applicability. The metrics for evaluating the model performance include accuracy, precision, recall, F1 score, area under receiver operating characteristic curve (AUC) and area under the precision-recall curve (AUPR). Each metric provides unique insights into different aspects of a model's performance: (1) Accuracy is the proportion of correctly classified instances among the total predictions. It is simple and easy to interpret but may not reflect class-specific performance and could overemphasize the majority class for imbalanced datasets. (2) Precision is useful when minimizing false positives is crucial; however, it ignores false negatives and thus not provide a complete model performance. (3) Recall emphasizes capturing all positive instances. There is a trade-off between recall and precision: increasing recall may decrease precision, and vice versa. (4) F1-score balances precision and recall and it is useful when both are important. It assumes equal importance of precision and recall and may not be suitable for all scenarios. (5) AUC is an important metric for binary classification and it assesses model performance across various decision thresholds. (6) AUPR is particularly useful in cases where the positive class (or the class of interest) is rare, making precision and recall more informative than accuracy but it may not be as interpretable as AUC. In order to ensure the stability of our proposed model, fivefold cross-validation (CV) was performed 10 times for CPIs task.

## Results and discussion

### Performance of SPVec-SGCN-CPI model using six corpuses

**Fig. 4:**
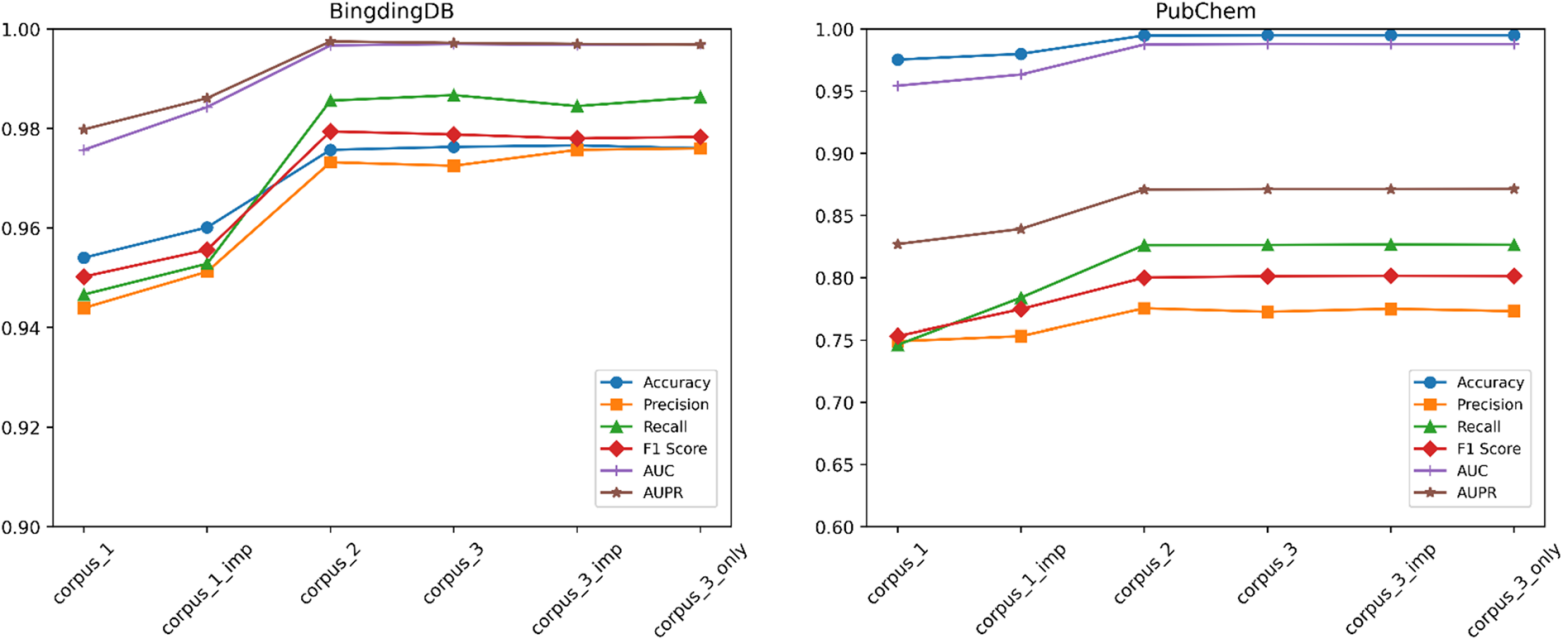
Classification performance of SPVec-SGCN-CPI model using six corpuses averaged over 10 runs on the BindingDB test set and PubChem test set

Figure 4 shows classification performance of SPVec-SGCN-CPI model averaged over 10 runs on two test sets. Although the model evaluation metrics achieved similar results using three corpuses on the training set (see Supplementary Figure S1), while increasing the size of the Corpus_1 to Corpus_2, SPVec-SGCN-CPI model achieved better performance on two test sets. Corpus_2 is larger encompassing a broader “vocabulary” and a more diverse range of “language” (i.e., protein sequences and SMILES) contexts, allowing the model to learn richer and more specific feature representations. Besides, larger corpus provides more contextual information, enabling the model to better understand the meanings of words in different contexts. This helps in generating word embeddings that are more contextually sensitive and enhance the model's generalizability. Test sets contained new ‘words’ not able to be represented by SPVec model and handling out-of-vocabulary “words” contributes to the descending prediction ability of the model. Compared to Corpus_1, replacing random vectors with averaged vectors of ‘words’ in Corpus_1_imp has improved the model performance, indicating that this approach is one of the measures to address the OOV problem. However, it's essential to note that the improvement in model performance is not always linear with the increase in corpus size. SPVec-SGCN-CPI model on Corpus_2 and Corpus_3 achieved roughly equivalent performance. This indicates that beyond a certain point, the marginal benefits of enlarging the corpus may diminish, while the computational requirements and training time increase. Moreover, Corpus_3 may contain noise or low-quality text, which could negatively impact the model. Corpus_3_imp after removing redundant data related to compounds and proteins did not experience a decline in model performance. This provides an option for removing noisy data associated with biological data. Corpus_3 and Corpus_3_only both achieved excellent model performance with no significant difference. The embeddings of proteins or compounds are obtained by summing and averaging each “word” (protein sequences and SMILES segments). We found that Corpus_3_only doesn’t contain any new “words”. SPVec has learned the optimized vector representation of each “word” even though not using new protein or new SMILES. Therefore, the inclusion of test set data in pre-training process by SPVec will not artificially inflate the model’s performance and generalization capability.

### Performance of SPVec compared with SPBert and SPGPT for feature representation

**Fig. 5:**
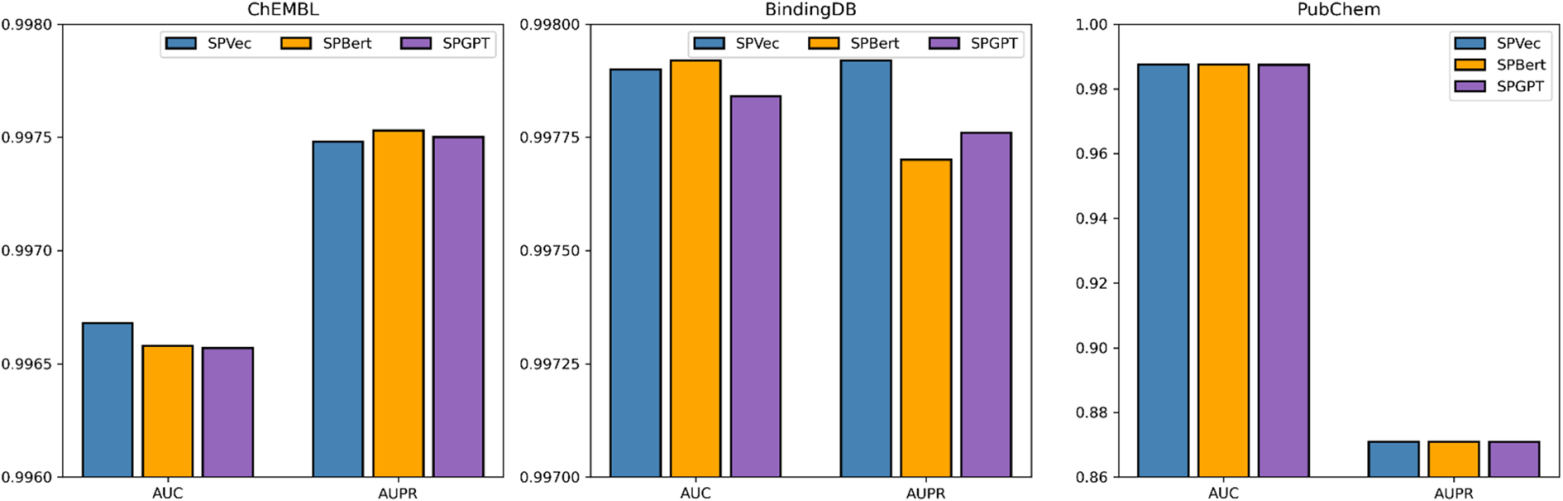
AUC and AUPR of three different feature representation methods on ChEMBL training set, BindingDB test set and PubChem test set

To demonstrate the feature representation capabilities of SPVec, we compared it with state-of-the-art large language models. We utilized bert-based pre-trained models, ChemBERTa-77M-MTR [59] and esm2_t33_650M_UR50D [60] for feature extraction. The combination of these is referred to as SPBert. Simultaneously, we employed GPT-based pre-trained models, ChemGPT-4.7M [61] and ProGPT2 [62] and their combination is denoted as SPGPT. We repeat the process 10 times for model evaluations on three datasets to reduce the influence of chance factors, thus improving the accuracy of our assessment of the model’s performance. Figure 5 illustrates the averaged AUC and AUPR while Figure S2 shows boxplot of AUC and AUPR with 10 repetitions using three different feature representation methods on ChEMBL (fivefold cross-validation dataset), BindingDB (test set), and PubChem (test set). All models performed well on fivefold cross validation and two independent testsets, suggesting saturation of modeling performance based on biological embeddings generated by pre-trained language models. This phenomenon is likely because the SGCN model's potent neighbor aggregation and topological graph representation capabilities for CPI information entail relatively basic feature requirements, and all three models can provide sufficient performance. Table 2 shows comparison of three feature representation methods in dimensionality, computation time, and memory consumption. For CPI prediction task, word2Vec might already suffice in capturing word semantics. Due to the higher dimensions ($d=1664$ for both SPBert and SPGPT) and substantial computational resource demands using SPBert and SPGPT, SPVec may become a more practical choice under resource constraints.

**Table 2:** Comparison of three feature representation methods in dimensionality, computation time and memory consumption

		SPVec		SPBert		SPGPT	
		SMILES2vec	ProtVec	ChemBERT	ESM2	ChemGPT	ProGPT2
Dimensionality		100	100	384	1280	384	1280
Feature generation	Time (Second)	137.11	49.39	1729.63	4688	1770.42	3549.28
	Memory Cost(GB)	2.88	0.56	1.21	4.27	1.48	12.53
Training process	Time (Hour)	2.43		5.03		4.87	
	Memory Cost(GB)	3.07		11.65		10.32	

All experiments are executed on Intel(R) Xeon(R) Gold 6230 CPU @ 2.10 GHz and the GeForce RTX 3080 was used to accelerate the training process

### Model performance and efficiency of different model combinations between SGCN and GCN

***Performance****.* Table 3 shows model performance of nine model combinations between SGCN and GCN on ChEMBL dataset. As the number (S)GCN of layers increases from 1 to 3, there is a consistent improvement in various performance metrics, suggesting that a larger receptive field or increased connectivity in the graph benefits the model's ability to make accurate predictions. When hop value gets larger, model performance barely improved. Figure S3 shows fivefold cross validation results averaged over 10 runs in the 1-hop to 4-hop setting. As the hop value from 1 increases to 3, there is a consistent improvement in various performance metrics, suggesting that a larger receptive field or increased connectivity in the graph benefits the model's ability to make accurate predictions. When hop value get larger, model performance improved little. We choose hop = 3 for following research. When the model utilizes only 3-order SGCN layers, it essentially undergoes linear transformation, that is a 3-hop neighbor aggregation. Its performance is superior to that of the 3-order GCN, indicating the unnecessity of non-linear transformations. Table 4 shows model performance of nine model combinations between SGCN and GCN on two independent test sets. Despite achieving comparable results on the training set, the SGCN model alone significantly outperforms the GCN and GCN + SGCN combinations on the test sets. This suggests that SGCN has advantages over GCN in terms of generalization. Specifically, the GCN model exhibits a decrease in performance on both test sets, indicating potential overfitting during training. On the PubChem dataset, SGCN consistently performs well across all layers, while GCN's performance is relatively limited. Notably, when the model consists of two or more layers, substituting the standard GCN layer with SGCN leads to an improvement in model performance. In summary, SGCN demonstrates superior generalization capabilities compared to GCN, particularly on independent test sets, and replacing GCN layers with SGCN layers can enhance model performance, especially in deeper architectures.

**Table 3:** Model performance of nine model combinations between SGCN and GCN on ChEMBL dataset

Model	Accuracy	Precision	Recall	F1-Score	AUC	AUPR
GCN	0.8940	0.9039	0.9166	0.9102	0.9557	0.9698
GCN + GCN	0.9462	0.9534	0.9548	0.9541	0.9868	0.9909
GCN + SGCN	0.932	0.9387	0.9459	0.9423	0.9802	0.9863
GCN + GCN + GCN	0.9757	0.9732	0.9856	0.9794	0.9967	0.9976
GCN + SGCN + SGCN	0.9459	0.9419	0.9680	0.9547	0.9861	0.9897
SGCN + SGCN + SGCN	0.9761	0.9742	0.9860	0.9776	0.9967	0.9975
GCN + GCN + SGCN	0.9754	0.9731	0.9854	0.9792	0.9966	0.9975
GCN + GCN + GCN + GCN	0.9871	0.9808	0.9935	0.9801	0.9972	0.9974
GCN + SGCN + GCN + SGCN	0.9752	0.9693	0.9860	0.9776	0.9967	0.9970

**Table 4:** Model performance of nine model combinations between SGCN and GCN on two independent test sets

	Model	Accuracy	Precision	Recall	F1-Score	AUC	AUPR
BindingDB	GCN	0.8493	0.8370	0.8669	0.8516	0.9261	0.9276
	GCN + GCN	0.8243	0.7966	0.8697	0.8316	0.9084	0.9065
	GCN + SGCN	0.8563	0.8426	0.8754	0.8587	0.9296	0.9273
	GCN + GCN + GCN	0.8386	0.8152	0.8748	0.8440	0.9201	0.919
	GCN + SGCN + SGCN	0.8691	0.8476	0.8993	0.8727	0.9433	0.9425
	SGCN + SGCN + SGCN	0.9805	0.9763	0.9847	0.9805	0.9979	0.9979
	GCN + GCN + SGCN	0.8394	0.8129	0.8808	0.8455	0.9202	0.9162
	GCN + GCN + GCN + GCN	0.8197	0.8066	0.8399	0.8229	0.8906	0.8989
	GCN + SGCN + GCN + SGCN	0.8618	0.8369	0.8938	0.8658	0.9375	0.9358
PubChem	GCN	0.8151	0.0419	0.6208	0.0785	0.7741	0.0558
	GCN + GCN	0.8246	0.0406	0.5666	0.0758	0.7676	0.0681
	GCN + SGCN	0.8436	0.0510	0.6433	0.0945	0.8147	0.0771
	GCN + GCN + GCN	0.8211	0.0407	0.6643	0.0856	0.7934	0.0701
	GCN + SGCN + SGCN	0.8380	0.0508	0.6659	0.0944	0.8331	0.1156
	SGCN + SGCN + SGCN	0.9948	0.7754	0.8262	0.8000	0.9875	0.8709
	GCN + GCN + SGCN	0.8031	0.0431	0.6840	0.0810	0.8010	0.0998
	GCN + GCN + GCN + GCN	0.8032	0.0401	0.5632	0.0743	0.7849	0.0695
	GCN + SGCN + GCN + SGCN	0.8364	0.0537	0.6623	0.0934	0.8321	0.1121

**Fig. 6:**
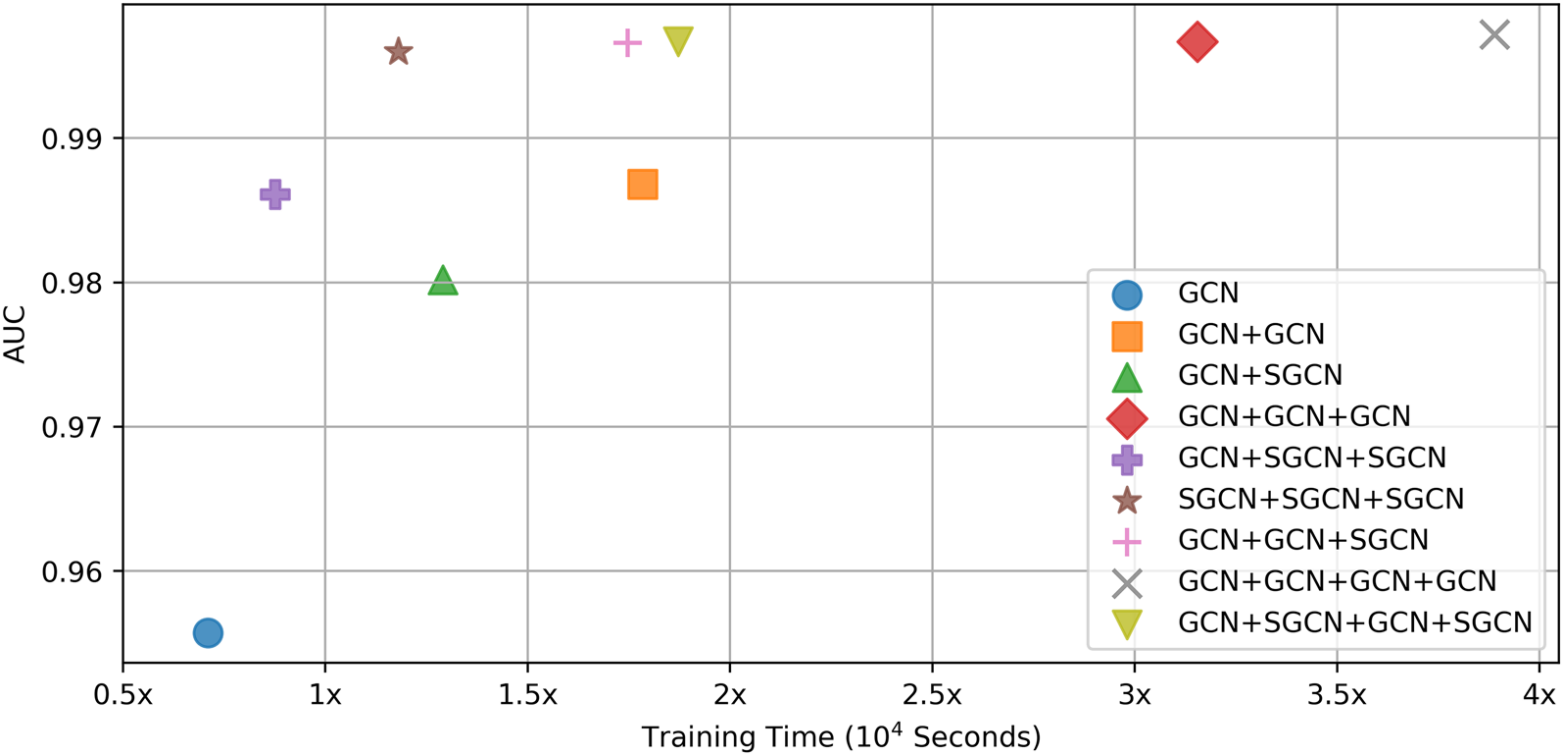
Training time and AUC of nine model combinations between SGCN and GCN

***Efficiency*.** Figure 6 illustrates the training time and AUC of nine model combinations between SGCN and GCN. As the standard GCN layer grows deeper, the training time increases gradually diminishing the model’s efficiency. However, utilizing SCN for $K$-order neighbor aggregation while simultaneously omitting non-linear layer-wise propagation can effectively reduce the model’s training time. For instance, comparing GCN + GCN + GCN and SGCN + SGCN + SGCN, the network structure of SGCN + SGCN + SGCN significantly enhances the model's training speed, reducing the training time by 72.23%, while still achieving competitive performance. Consequently, substituting the standard GCN layer with SGCN can effectively extends the layers of the GCN network without the occurrence of neighbor explosion and improve the training speed on the premise of ensuring the model efficiency.

### Performance of SPVec-SGCN model compared with ML- and DL-based models on BindingDB and PubChem test sets

On one hand, accuracy, precision, recall, F1-score, AUC and AUPR of SPVec-SGCN model on BindingDB testset reached 0.9805, 0.9763, 0.9847, 0.9805, 0.9979 and 0.9979 respectively. On the other hand, accuracy, precision, recall, F1-score, AUC and AUPR of SPVec-SGCN model on PubChem testset reached 0.9948, 0.7754, 0.8262, 0.8000, 0.9875 and 0.8709 respectively. These results demonstrate its extraordinary predictive ability for CPI prediction tasks, especially in unbalanced data. We compared the SPVec-SGCN model with Gaussian Naïve Baysian (GNB), RF, GBDT and DNN to show its superiority in CPI prediction. Our proposed model is superior to other traditional ML and DL models on training set (Table S4) and two test sets (Table 5). On BindingDB test set, The AUCs of our method are higher than GNB, RF, GBDT and DNN by 46.27, 14.11, 29.04, and 21.37%, respectively. All four ML and DL models failed to predict CPIs on PubChem test set, because these models may have a bias towards the majority class, struggling to identify or distinguish the minority class properly. For instance, GBDT and DNN overlooked minority samples and predicted almost the entire sample to negative. The excellent performance of our model is attributed to its effective and powerful graph processing ability via adaptive neighbor feature aggregation. The results show that our model has learned robust patterns and features that are applicable across various data distributions, rather than being overfit to a specific dataset. Generalizability and robustness of our model have been validated.

**Table 5:** Results of SPVec-SGCN model performance compared with machine learning- and deep learning-based models on BindingDB and PubChem test sets

Testset	Methods	Accuracy	Precision	Recall	F1-Score	AUC	AUPR
BindingDB	Ours	0.9805	0.9763	0.9847	0.9805	0.9979	0.9979
	GNB	0.6354	0.6236	0.6789	0.6500	0.6822	0.6615
	RF	0.7747	0.8072	0.7205	0.7614	0.8689	0.8745
	GBDT	0.6868	0.6524	0.7964	0.7172	0.7751	0.7733
	DNN	0.751	0.7412	0.7694	0.755	0.8222	0.807
PubChem	Ours	0.9948	0.7754	0.8262	0.8000	0.9875	0.8709
	GNB	0.5954	0.1558	0.5757	0.3708	0.6070	0.1672
	RF	0.8246	0.0466	0.6460	0.0835	0.7648	0.1940
	GBDT	0.5538	0.0207	0.7359	0.0402	0.6949	0.0244
	DNN	0.6003	0.0202	0.6411	0.0391	0.6374	0.0179

### Further experimentation with imbalanced datasets

**Fig. 7:**
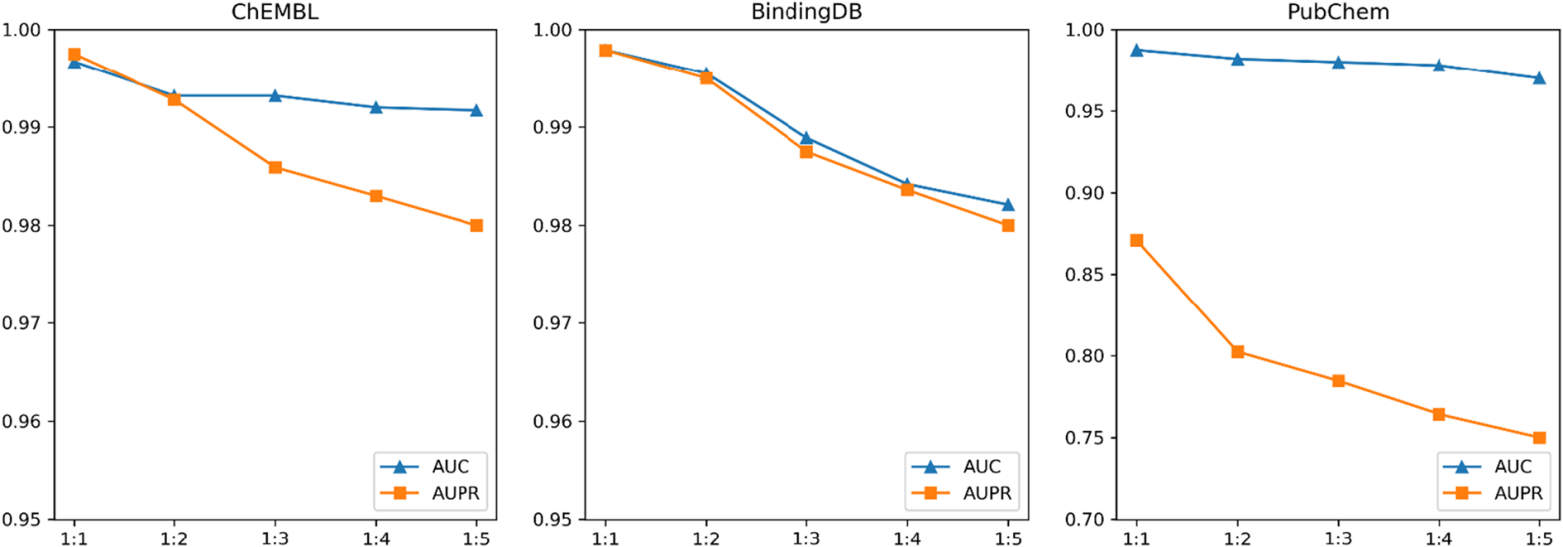
the model performance of our model under different proportions of positive and negative samples on ChEMBL training set, BindingDB test set and PubChem test set

Besides compound-protein pairs with IC50 > 10000 $nM$, we randomly selected the matching number of the unknown compound-protein pairs (by excluding all known CPIs) as negative samples [50, 51]. Figure 7 shows the model performance of our model under different proportions of positive and negative samples on ChEMBL training set, BindingDB test set and PubChem test set. As the proportion of positive and negative samples increases, the AUC remains unchanged on the model training set. Due to changes in the balance between precision and recall, AUPR is more sensitive to class imbalance, resulting in a slight decrease in AUPR. On the BindingDB and PubChem test sets, as the proportion of positive and negative samples increases, both AUC and AUPR values show a decreasing trend. This indicates that with the increase of negative samples, the performance of the model in the entire sample space decreases slightly. However, even at a positive-to-negative sample ratio of 1:5, both AUC and AUPR remain high, indicating that the model performs well in handling class imbalance issues. The model's high AUPR may also suggest its strong ability to identify positive instances (minority classes), meaning that it can find true positive instances while maintaining a low misclassification rate.

### Further experimentation with larger datasets and deeper SGCN

**Fig. 8:**
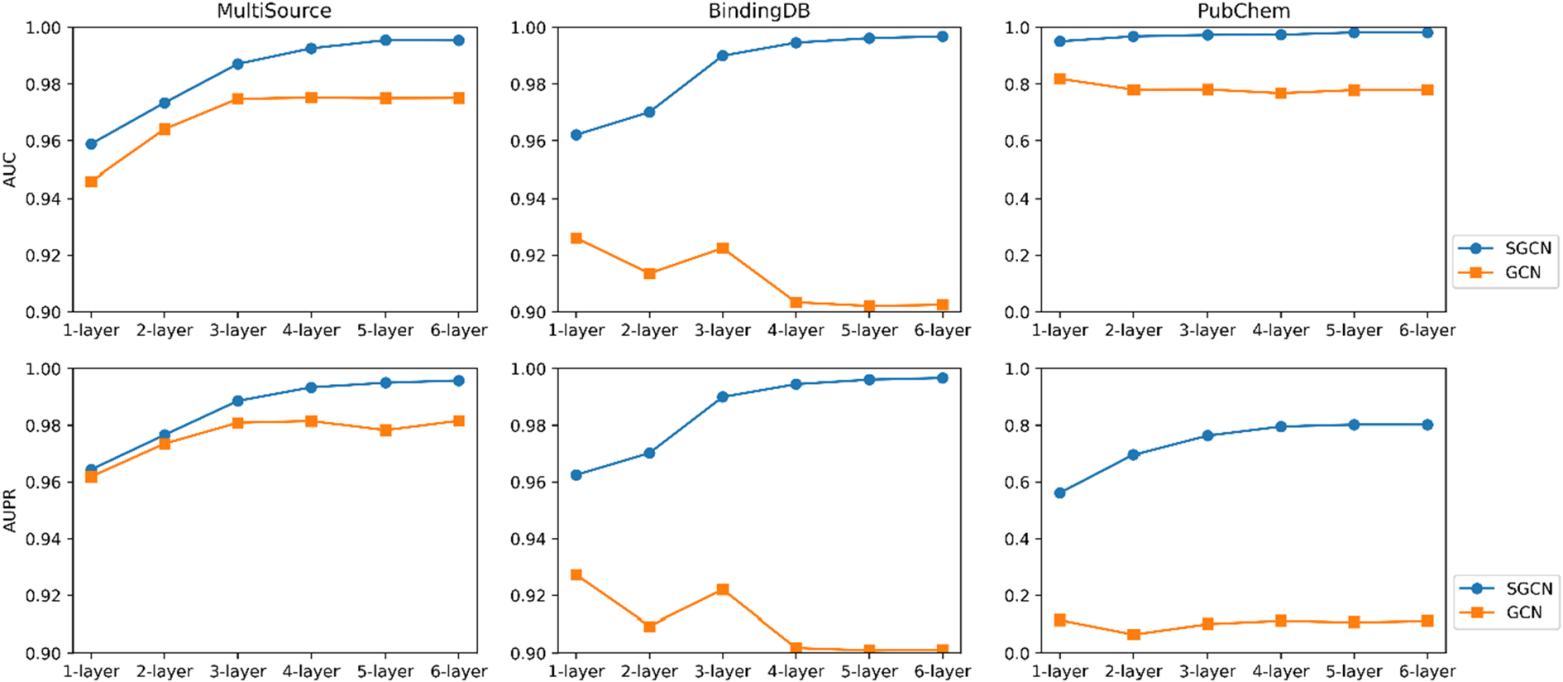
Model performance of SGCN and GCN models with different layers on MultiSource training set, BindingDB testset and PubChem testset averaged over 10 runs

To better showcase the superiority of the SGCN model, we collected data from multiple sources to augment the dataset. Details of the augmented training set (named MultiSource) can be found in Table S5. After data cleaning, there are a total of 676,414 positive samples and 319,197 negative samples in the MultiSource dataset, with a ratio of approximately 2:1. Deeper SGCN models were employed on this larger dataset. Figure 8 illustrates model performance of SGCN and GCN models with different layers on MultiSource training set, BindingDB testset and PubChem testset averaged over 10 runs. It is important to note that data from the BindingDB and PubChem test sets were not involved in the model fine-tuning process.

As the number of layers in the SGCN and GCN increases from 1 to 5, the performance shows a gradual improvement on the MultiScource dataset. This suggests that adding layers contributes to the models learning more complex representations, thereby enhancing performance. When the number of layers reaches 5, the model reaches its optimum. With further increases in the number of layers, the performance of the model no longer improves. Across all layers, SGCN generally outperforms GCN in terms of both AUC and AUPR indicating that the SGCN is more effective on imbalanced training data. In the BindingDB dataset, as the number of layers increases, the SGCN model exhibits a similar trend to that observed in the MultiSource dataset. However, the GCN model shows a decline in model performance after 3 layers, indicating the occurrence of overfitting. On the PubChem dataset, SGCN performs well across all layers, while GCN's performance is relatively limited.

### Performance of SPVec-SGCN model compared with six state-of-the-art models

In order to provide additional evidences of the efficacy of our proposed SPVec-SGCN-CPI approach, we conducted a comparative analysis with six other existing state-of-the-art methods published. Below are brief descriptions of each of these methods. (1) PMFCPI [63] is a pre-trained multi-functional model with assessing drug selectivity. (2) GraphCPI [64] is a novel graph-based computational model for potential CPIs. (3) STCPI [65] is self-training model with augmenting negative samples. (4) GcForest [66] is an ensemble decision tree learning algorithm with unique features. (5) CCL-DTI [67] contributes the contrastive loss in CPI prediction using CNN. (6) SgCPI [68] is heterogeneous sampled subgraph neural networks model with knowledge distillation. The results in Table 6 demonstrate that SubSGCN-CPI outperforms the four state-of-the-art methods in terms of performance evaluation metrics on BindingDB and PubChem test sets. Although these state-of-the-art methods achieved relatively high performance on training phase (Table S6), our method exhibits superior performance with higher scores across all metrics for the BindingDB dataset. Accuracy (0.9805), precision (0.9763), recall (0.9847), F1-Score (0.9805), AUC (0.9979), and AUPR (0.9979) are higher than second best model (i.e., PMFCPI) by 19.31, 19.21, 15.83, 19.08, 11.35, 10.34%. Our method also demonstrates superior performance on PubChem dataset displaying highest scores in AUPR (0.8709), which is 20.22% higher than STCPI. PMFCPI, GcForest and GraphCPI achieved notably lower precision scores, showing poor ability to predict unbalanced data. These results suggest that SPVec-SGCN model is an effective approach to enhance the accuracy of CPI prediction. SPVec-SGCNs combined beneficial similarity features to build a homogeneous network, thereby maximizing the utility of available information through the aggregation of neighborhood data. And, SPVec-SGCNs utilized three-layer simplified GCN model to ensure parameters of graph structure which offers advantages in terms of accuracy, scalability and training speed.

**Table 6:** Classification results of SPVec-SGCNs model compared with four state-of-the-art models on BindingDB and PubChem test sets

Testset	Methods	Accuracy	Precision	Recall	F1-Score	AUC	AUPR
BindingDB	Ours	0.9805	0.9763	0.9847	0.9805	0.9979	0.9979
	PMFCPI	0.8218	0.8189	0.8501	0.8234	0.8962	0.9044
	GraphCPI	0.7237	0.7478	0.7016	0.7223	0.7697	0.7734
	STCPI	0.8234	0.7965	0.7999	0.8228	0.8752	0.8745
	GcForest	0.862	0.8523	0.8547	0.8678	0.8956	0.8957
	CCL-DTI	0.8749	0.8594	0.8782	0.8631	0.9021	0.8954
	SgCPI	0.8334	0.8348	0.8329	0.8335	0.8521	0.8545
PubChem	Ours	0.9948	0.7754	0.8262	0.8000	0.9875	0.8709
	PMFCPI	0.6967	0.1893	0.6743	0.4461	0.7880	0.2243
	GraphCPI	0.6253	0.0587	0.6227	0.1048	0.7653	0.2540
	STCPI	0.8439	0.6261	0.7359	0.6007	0.8949	0.7244
	GcForest	0.6003	0.0202	0.6411	0.0391	0.6374	0.2037
	CCL-DTI	0.6482	0.1467	0.5649	0.3732	0.8036	0.1734
	SgCPI	0.8679	0.0573	0.6557	0.1064	0.8278	0.0693

### Prediction and validation of unidentified CPIs

**Fig. 9:**
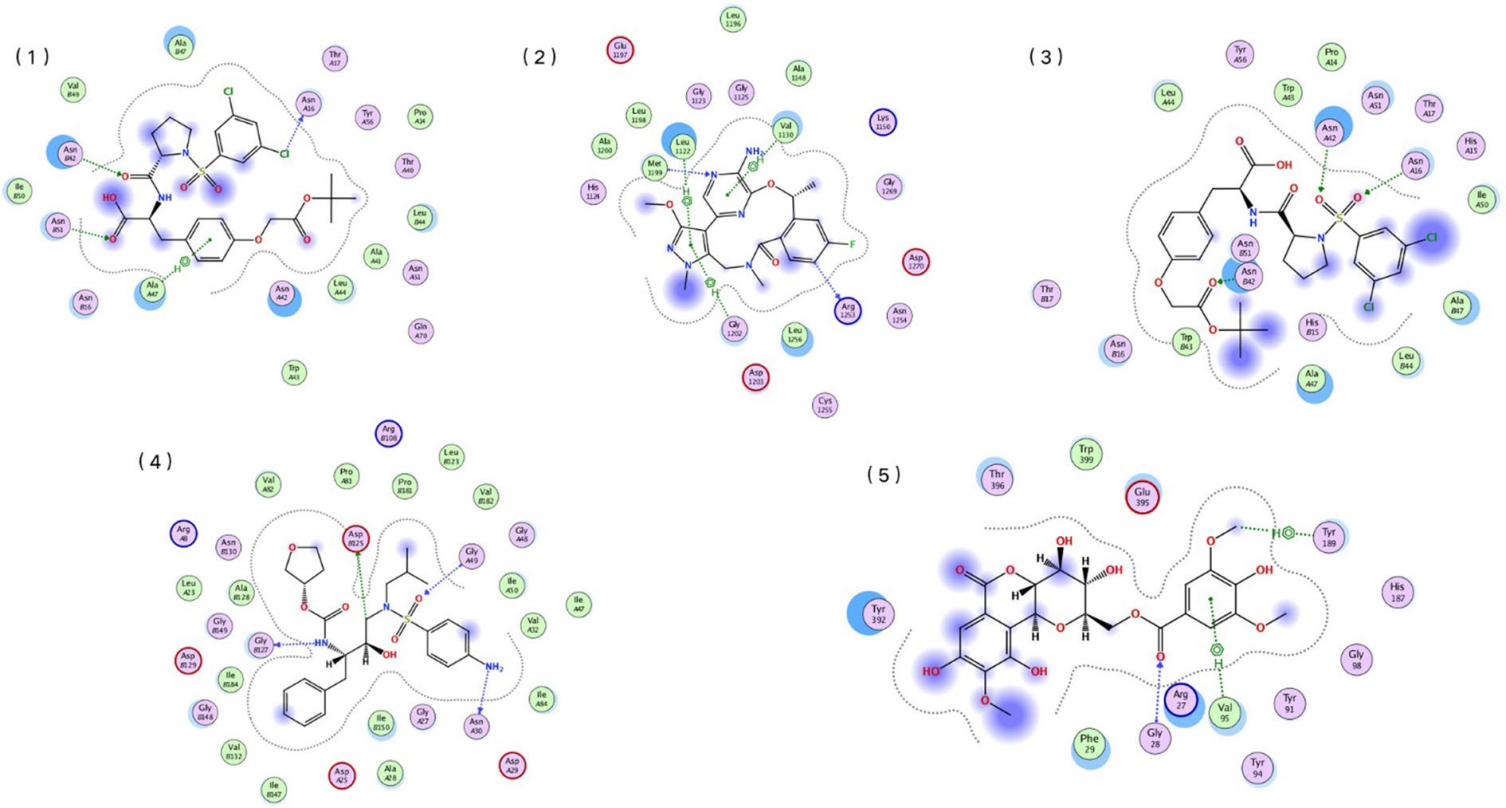
Interactions of top five ranked compounds-protein pairs predicted by SPVec-SGCN model via molecular docking

To further validate the CPIs prediction ability of SPVec-SGCN model, we scored all the unlabeled CPIs on the ChEMBL dataset. Table S7 lists top 30 predicted CPIs. Specifically, we identified the top five ranked CPIs by molecular docking (MD) and cross-referenced them with external supporting evidences from relevant databases and biomedical literature. Figure 9 shows interactions of top five ranked compounds-protein pairs via MD and Table S8 shows the positions, bond types, distances, and energy values of the interaction relationships among top five ranked compounds-protein pairs, which is important to understand mechanisms of CPIs. All five compound-protein pairs have various interactions like H-bond and Vanderwals force. Table 7 shows detailed information and external supporting evidence of top five ranked compounds-protein pairs. Except for the third compound protein pair, all of them have very low Ki or IC50 values, which is also consistent with the results of molecular simulations, demonstrating a strong interaction between compound and target protein. However, the third compound-protein pair has a higher IC50 value because our training and modeling process is based on the premise that similar compounds and protein targets are related and similar compound-protein pairs tend to have similar interactions, as described above. However, protein-related life activities are complex and do not fully conform to this assumption. We examined the training data and found that there was a very strong interaction (IC50: 0.860 nM) between the third ranked molecule and another protein that was highly similar (sequence identity: 99%) to the predicted target (Integrin alpha-4/beta-7), which was responsible for the high prediction score. Overall, these results suggest that the SPVec-SGCN-CPI model is highly effective in predicting novel CPIs and has important potential in drug discovery and development.

**Table 7:** Detailed information and external supporting evidence of top five ranked compounds-protein pairs predicted by SPVec-SGCN model

Rank	Compounds	Target Name	Bioactivity data	References
1	CHEMBL823551	Integrin alpha-4/beta-7	IC50: 211 nM	[69]
2	CHEMBL3286826	ALK tyrosine kinase receptor/Nucleophosmin	Ki: < 0.0800 nM	[70]
3	CHEMBL345144	Integrin alpha-4/beta-7	IC50: 2.10E + 3 nM	[71]
4	CHEMBL116	Protease	Ki: 0.00700 nM	[72]
5	CHEMBL1120718	Histamine H3 receptor	Ki:0.3 nM	[73]

## Conclusion

In this study, we proposed an end-to-end approach, named SPVec-SGCN-CPI, which utilizes simplified GCN model information based on low-dimensional and continuous feature generated by SPVec model and graph topology information for predicting CPIs. The SGCN technique separated local neighborhood aggregation step and nonlinearity layer-wise propagation step to effectively aggregate $K$-order neighbor information under the premise of avoiding neighbor explosion and accelerating training. This makes the method more effective than other traditional methods that can't handle such complexity. The study evaluated the performance of SPVec-SGCN-CPI method on the three databases and compared it with classic ML and DL methods such as GNB, GBDT, RF, and DNN, as well as advanced CPI prediction methods, including PMFCPI, GraphCPI, STCPI and GcForest. The classification results illustrated that SPVec-SGCN-CPI outperformed all these methods in terms of prediction accuracy, especially on unbalanced data. SPVec-SGCN-CPI is capable of propagating node features and topological information to the feature space, which enables the method to take interactions among CPIs into account for fusion of heterogeneity. All unlabeled data in ChEMBEL were scored using our method and top five ranked CPIs were confirmed by molecular docking and existing evidence. The results suggest that our model can discover reliable CPIs among unlabeled compounds-protein pairs. This discovery has significant implications for drug re-profiling and drug discovery. Overall, SPVec-SGCN has demonstrated its superior ability to predict CPIs. This method has great potential to contribute to the identification of new targets and improve the efficiency of drug discovery.

### Supplementary Information

Supplementary Material 1.

## Data Availability

All source codes are available in the GitHub repository https://github.com/yufangz-sjtu/SPVec-SGCN-CPI. Furthermore, the raw data used in this work can be downloaded from https://www.bindingdb.org/rwd/bind/chemsearch/marvin/SDFdownload.jsp?download_file=/bind/downloads/BindingDB_All_2D_202310_sdf.zip. The processed datasets and features extracted by our method can be downloaded from https://pan.quark.cn/s/f5c39d786029 (password: aUPW).

## Abbreviations

CPIs: Compound-protein interactions
ML: Machine learning
DL: Deep learning
RF: Random Forest
SVM: Support Vector Machine
DNN: Deep Neural Network
GBDT: Gradient Boosting Decision Tree
GCN: Graph convolutional network
CNN: Convolutional neural network
MLP: Multilayer perceptron
SGCN: Simplified Graph convolutional network
AUC: Area under receiver operating characteristic curve
AUPR: Area under the precision-recall curve
CV: Cross-validation
GNB: Gaussian Naïve Baysian
MD: Molecular docking
