# Supplementary material for: An end-to-end method for predicting compound-protein interactions based on simplified homogeneous graph convolutional network and pre-trained language model
Source: J Cheminform. 2024 Jun 7;16:67. doi: 10.1186/s13321-024-00862-9 (PMC11162000; doi:10.1186/s13321-024-00862-9)
Supplement: Supplementary file 1 — Supplementary Material 1. [file 13321_2024_862_MOESM1_ESM.docx]

# An end-to-end method for compound-protein interaction based on simplified homogeneous graph convolutional network and pre-trained language model

Yufang Zhang^1,2,3^, Jiayi Li^4^, Shenggeng Lin^4^, Jianwei Zhao^4^, Yi Xiong^4,5*^and Dong-Qing Wei^2,3,4*^

^1^School of Mathematical Sciences and SJTU-Yale Joint Center for Biostatistics and Data Science,

Shanghai Jiao Tong University, Shanghai 200240, China

^2^Peng Cheng Laboratory, Shenzhen, Guangdong 518055, China

^3^Zhongjing Research and Industrialization Institute of Chinese Medicine, Zhongguancun Scientific Park, Meixi, Nanyang, Henan, 473006, China

^4^State Key Laboratory of Microbial Metabolism, School of Life Sciences and Biotechnology, and

Joint Laboratory of International Cooperation in Metabolic and Developmental Sciences, Ministry of

Education, Shanghai JiaoTong University, Shanghai, China

^5^Shanghai Artificial Intelligence Laboratory, Shanghai, 200232, China

*Corresponding author: xiongyi@sjtu.edu.cn; dqwei@sjtu.edu.cn

**Table S1** Numbers of compounds, target and their interactions from three datasource as of October 2023.

| Datasource | Compounds | Targets | Interactions |
| --- | --- | --- | --- |
| ChEMBL | 617972 | 4506 | 1503403 |
| BindingDB | 47159 | 1998 | 89070 |
| PubChem | 55741 | 603 | 120567 |


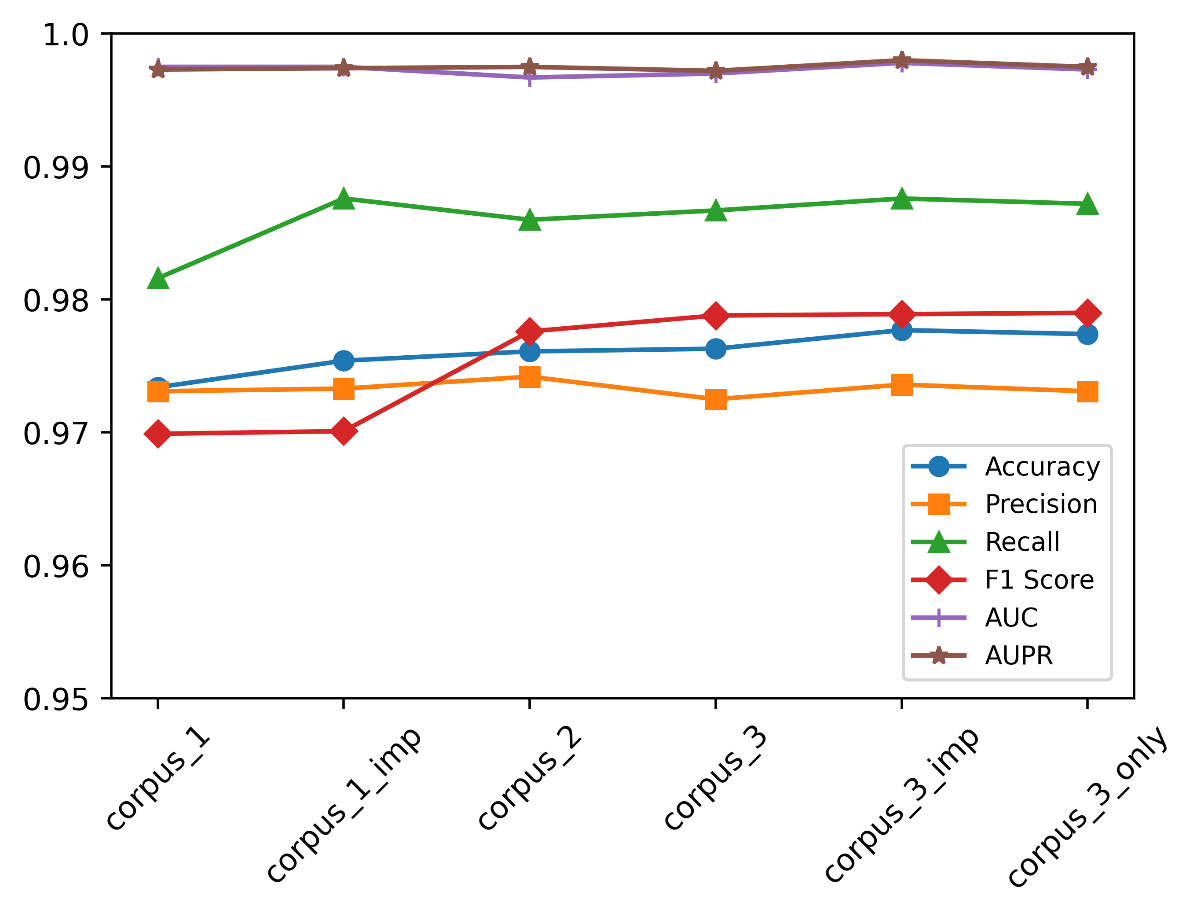


**Figure S1** Classification performance of SPVec-SGCN-CPI model using six corpuses by 5-fold cross validation averaged over 10 runs on the training dataset.

**Table S2** Different threshold values adopted by researchers in compound-protein (or drug-target) interaction prediction task

| References | Datasets | Index | Threshold of positive samples | Threshold of negative samples |
| --- | --- | --- | --- | --- |
| Zheng [1]  Wang [2] | Human dataset,  BindingDB | IC50 | <100 *n*M | >10^4^ *n*M (10μM) |
| Chatterjee [3] | DrugBank, BindingDB, DTC | $K_{i}$, $K_{d}$, IC50, and EC50 | ≤10^3^ *n*M nM(1.0μM ) | ≥10^6^ *n*M  (1000μM) |
| Lim [4] | ChEMBL, DUD-E,  PDB-bind | IC50 | <1.0 μM | >1.0 μM |
| Wen [5] | ChEMBL, DUD-E, MUV | IC50 | <50μM | >50 μM |
| Zeng [6, 7] | ChEMBL,  DrugCentral | $K_{i}$, $K_{d}$, IC50 or EC50 | ≤10 μM | >10 μM |
| Rifaioglu[8] | ChEMBL | IC50, $K_{i}$, $K_{d}$, potency.. | ≤10 μM | ≥20 μM |
|  |  | IC50,$K_{i}$,EC50 | ≤10 μM | >10μM |
| Lee [9] | PubChem | $K_{d}$ | < 10μM | >10μM |
| Huang [10] | KinaseSARfari,  IUPHAR, PubChem BioAssay | $K_{d}$ | < 10μM | >10μM |
| Gao [11] | DrugBank Davis  KIBA | $K_{d}$ | <5μM | ≥ 5μM |

While other measures such as AC50, EC50 are also important in characterizing CPIs, the IC50 or $K_{i}$/$K_{d}$ has been widely used in pharmacology and drug discovery to quantify the potency of compounds in inhibiting the activity of a biological target. We know that the determination of IC50 only requires drawing a gradient of inhibitor concentrations to fit the growth curve. IC50 is most commonly reported in experimental studies because determination process of $K_{i}$ is slightly cumbersome, even though $K_{i}$ is a more accurate measurement index. There are 1,031,500 entries of CPI with known IC50 values 375,933 entries with known $K_{i}$ values and only 84,874 entries with known Kd values in three datasets. Additionally, most records with $K_{i}$ or $K_{d}$ values also provide IC50 data. We focused on binary classification to predict CPIs, which accurate binding affinity values are not that important. Either a low IC50 value or a low $K_{i}$/$K_{d}$ value indicates high binding affinity. In some cases, IC50 ≈ $K_{i}$ = $K_{d}$ [12]. In order to maximize the utilization of our collected data, we selected IC50 as the primary quantitative measure. By following the activity threshold discussion in [1,2], compound-protein pairs with IC50 values <100 nM were selected as positive samples and compound-protein pairs with IC50 values >10000 nM were selected as negative samples. It is worth noting that this threshold is variable. It can adjust the IC50 value [4,5] or classify positive and negative samples based on $K_{i}$/$K_{d}$ values [9-11] and all bioassay data [3,6-8].

**Table S3** Detailed information about hyperparameters and architectures of SGCN model.

| network | dimensions | 512 |
| --- | --- | --- |
|  | aggregatation | concat |
|  | loss | sigmoid |
|  | architecture | three layers: SGCN-SGCN-SGCN |
|  | activation | Relu |
|  | bias | norm |
| parameters | learning rate | 0.001 |
|  | dropout | 0.1 |
|  | weight decay | 0.0 |
|  | sample coverage | 50 |
|  | positive weight | 1 |
|  | epoch | 1000 |


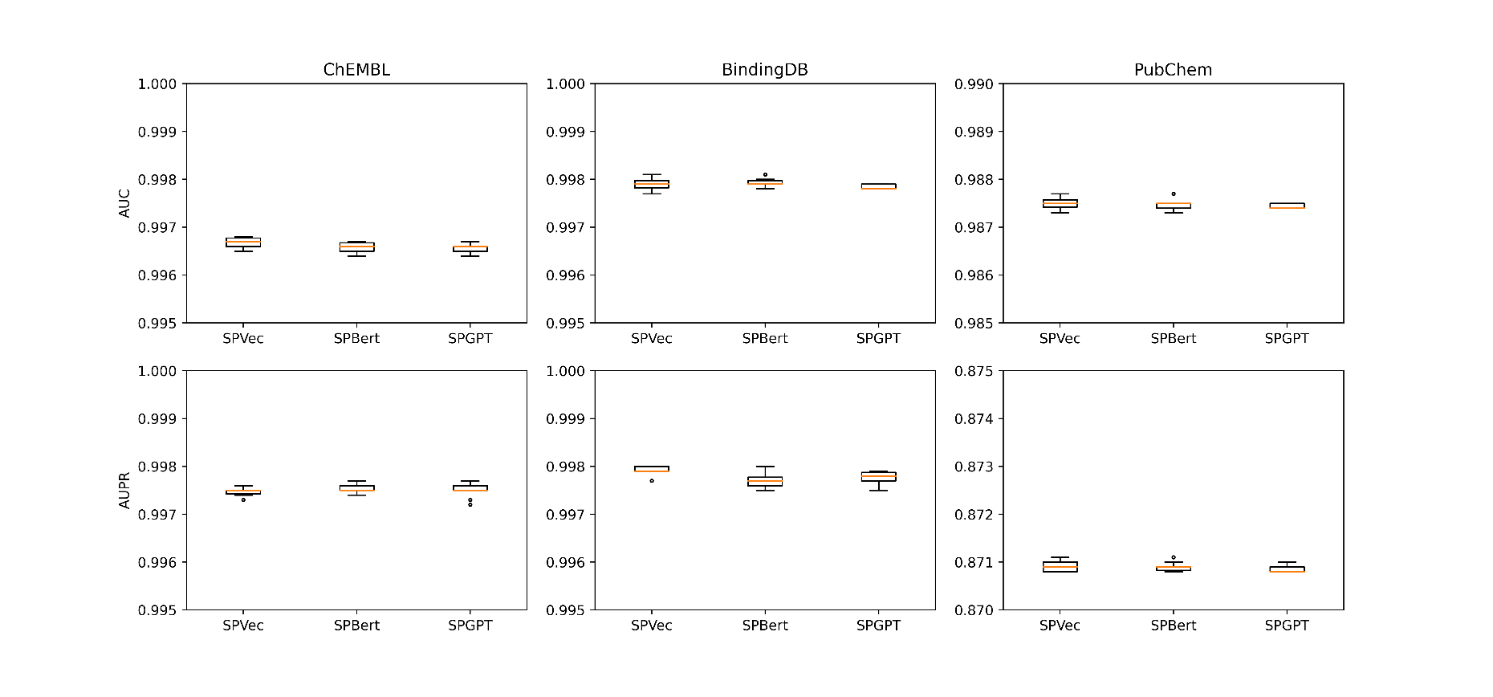


Figure **S2.** AUC and AUPR with 10 repetitions of three different feature representation methods on ChEMBL training set, BindingDB test set and PubChem test set.

**
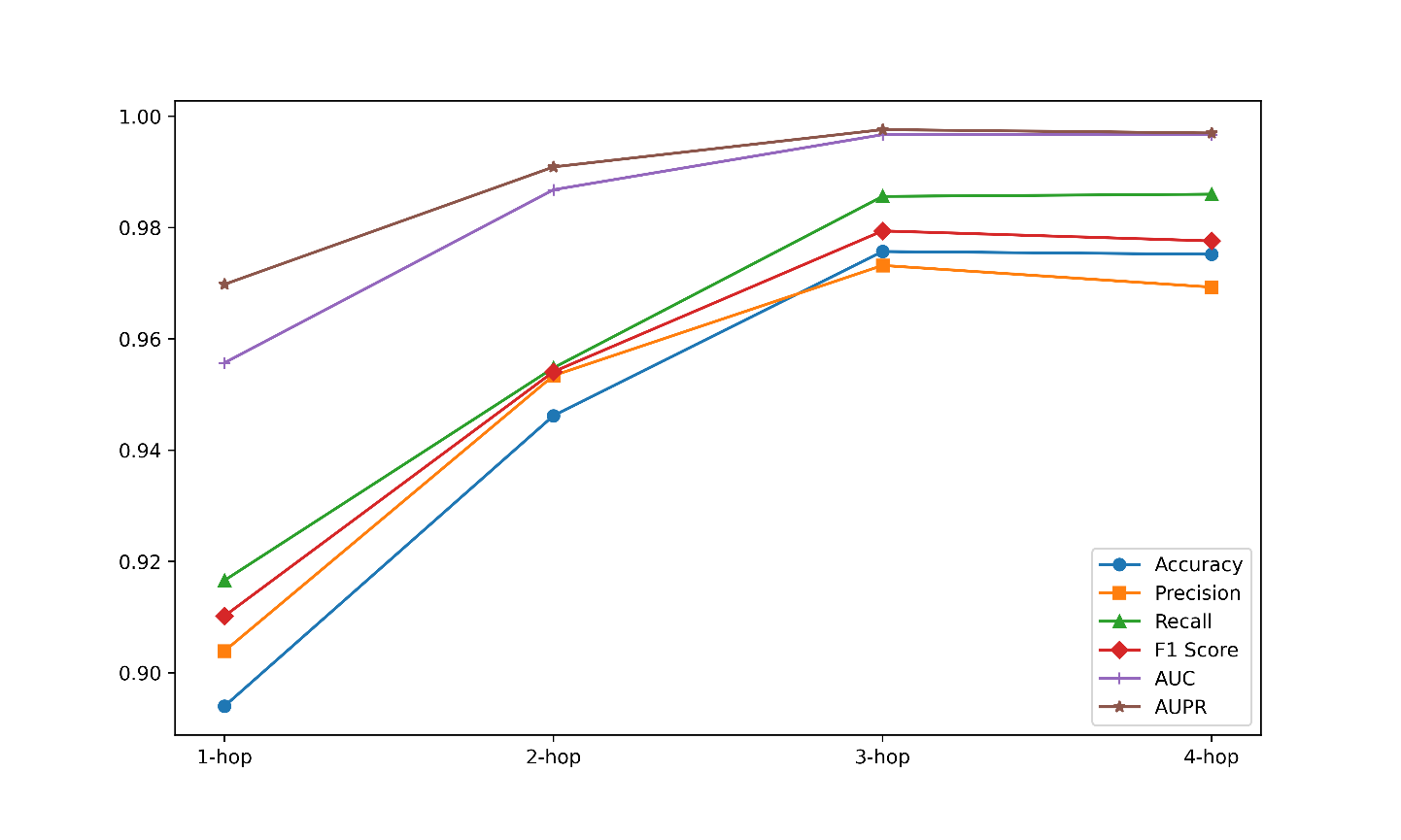
**

**Figure S3** 5-fold cross validation results averaged over 10 runs in the 1-hop to 4-hop setting.

**Table S4** 5-fold cross validation results averaged over 10 runs of SGCN-CPI performance compared with classic machine learning and deep learning models on ChEMBEL dataset.

| **Methods** | **Accuracy** | **Precision** | **Recall** | **F1-Score** | **AUC** | **AUPR** |
| --- | --- | --- | --- | --- | --- | --- |
| **Ours** | **0.9761** | **0.9742** | **0.9860** | **0.9776** | **0.9967** | **0.9975** |
| GNB | 0.6640 | 0.7048 | 0.7480 | 0.7257 | 0.7103 | 0.7734 |
| RF | 0.9118 | 0.9108 | 0.9432 | 0.9267 | 0.9662 | 0.9744 |
| GBDT | 0.7674 | 0.7620 | 0.8853 | 0.8190 | 0.8402 | 0.8730 |
| DNN | 0.8964 | 0.9081 | 0.9188 | 0.9134 | 0.9499 | 0.9592 |

**Table** **S5**. Details of the augmented training set (named MultiSource)..

| Datasource | Compounds | Targets | Interactions |
| --- | --- | --- | --- |
| ChEMBL | 617972 | 4506 | 1503403 |
| US Patent | 486751 | 2171 | 1034910 |
| PDSP Ki | 3301 | 399 | 27733 |
| Taylor Research Group UCSD | 50 | 5 | 274 |
| CSAR | 622 | 6 | 818 |
| WIPO | 4250 | 60 | 5961 |
| D3R | 2226 | 21 | 2990 |

**Table S6** 5-fold cross validation results averaged over 10 runs of SGCN-CPI performance compared with four state-of-art models on ChEMBEL dataset.

| **Methods** | **Accuracy** | **Precision** | **Recall** | **F1-Score** | **AUC** | **AUPR** |
| --- | --- | --- | --- | --- | --- | --- |
| **Ours** | **0.9761** | **0.9742** | **0.9860** | **0.9776** | **0.9967** | **0.9975** |
| PMFCPI | 0.9661 | 0.9165 | 0.9473 | 0.9289 | 0.984 | 0.9828 |
| GraphCPI | 0.9706 | 0.9783 | 0.9801 | 0.9774 | 0.9931 | 0.9932 |
| STCPI | 0.9774 | 0.9702 | 0.9854 | 0.9709 | 0.9962 | 0.9960 |
| GcForest | 0.9659 | 0.9715 | 0.9774 | 0.9765 | 0.9829 | 0.9821 |
| CCL-DTI | 0.9716 | 0.9722 | 0.9789 | 0.9782 | 0.9867 | 0.9856 |
| SgCPI | 0.9634 | 0.9625 | 0.9726 | 0.972 | 0.9734 | 0.9783 |

**Table S7** Top 30 predicted compounds-protein interactions predicted by our model.

| Compounds | Target Name |
| --- | --- |
| Clc1cc(Cl)cc(S(=O)(=O)N2[C@H](C(=O)N[C@H](C(=O)O)Cc3ccc(OCC(=O)OC(C)(C)C)cc3)CCC2)c1 | Integrin alpha-4/beta-7 |
| COc1nn(C)c2CN(C)C(=O)c3ccc(F)cc3[C@@H](C)Oc3nc(cnc3N)-c12 | ALK tyrosine kinase receptor/Nucleophosmin |
| Clc1cc(Cl)cc(S(=O)(=O)N2[C@H](C(=O)N[C@H](C(=O)O)Cc3cnccc3)CCC2)c1 | Integrin alpha-4/beta-7 |
| S(=O)(=O)(N(C[C@@H](O)[C@@H](NC(=O)O[C@@H]1COCC1)Cc1ccccc1)CC(C)C)c1ccc(N)cc1 | Protease |
| O=C1C=CN(c2ccc(-c3nc4c(cc(CCN5[C@H](C)CCC5)cc4)cc3)cc2)C=C1 | Histamine H3 receptor |
| Clc1cc(Cl)cc(S(=O)(=O)N2[C@@](C(=O)N[C@H](C(=O)O)Cc3ccccc3)(C)CCC2)c1 | Integrin alpha-4/beta-7 |
| Clc1cc(Cl)cc(S(=O)(=O)N2[C@H](C(=O)N[C@H](C(=O)O)Cc3ccc(OCCCC)cc3)CCC2)c1 | Integrin alpha-4/beta-7 |
| Clc1cc(Cl)cc(S(=O)(=O)N2[C@H](C(=O)N[C@H](C(=O)O)Cc3ccc(OCc4ccccc4)cc3)CCC2)c1 | Integrin alpha-4/beta-7 |
| Clc1cc(Cl)cc(S(=O)(=O)N2[C@H](C(=O)N[C@H](C(=O)O)Cc3ccc(O)cc3)CCC2)c1 | Integrin alpha-4/beta-7 |
| Clc1cc(Cl)cc(S(=O)(=O)N2[C@H](C(=O)N[C@H](C(=O)O)Cc3ccc(OC)cc3)CCC2)c1 | Integrin alpha-4/beta-7 |
| S(=O)(=O)(c1ccccc1)C1(C(=O)N[C@H](C(=O)O)Cc2ccc(-c3c(OC)cccc3)cc2)CCN(C(C)(C)C)CC1 | Integrin alpha-4/beta-7 |
| S(=O)(=O)(N(C)C)C1CCN(c2nc(-n3c(C(F)F)nc4c(OC)cccc34)nc(N3CCOCC3)n2)CC1 | Phosphatidylinositol 3-kinase regulatory subunit alpha/4 5-bisphosphate 3-kinase catalytic subunit beta isoform |
| Clc1cc(Cl)cc(S(=O)(=O)N2[C@H](C(=O)N[C@H](C(=O)O)Cc3ccc(-c4c(OC)cccc4)cc3)CSC2)c1 | Integrin alpha-4/beta-7 |
| S(=O)(=O)(N(C)C)C1CCN(c2nc(-n3c(C(F)F)nc4c(OC)cccc34)nc(N3CCOCC3)n2)CC1 | Phosphatidylinositol 3-kinase regulatory subunit alpha/4 5-bisphosphate 3-kinase catalytic subunit delta isoform |
| Clc1cc(Cl)cc(S(=O)(=O)N2[C@H](C(=O)N[C@H](C(=O)O)Cc3ccc(-c4c(OC)cccc4)cc3)CCC2)c1 | Integrin alpha-4/beta-7 |
| S(=O)(=O)(C)N1CCN(c2nc(-n3c(C(F)F)nc4c(OC)cccc34)nc(N3CCOCC3)c2)CC1 | Phosphatidylinositol 3-kinase regulatory subunit alpha/4 5-bisphosphate 3-kinase catalytic subunit beta isoform |
| Clc1c(Nc2c(S(=O)(=O)N(C)C)cccc2)nc(Nc2c(OC)cc(N(CCN(C)C)C)c(NC(=O)C=C)c2)nc1 | ALK tyrosine kinase receptor/Echinoderm microtubule-associated protein-like 4 |
| Clc1ccc([C@@]2(C(=O)N[C@H](C(=O)O)Cc3ccc(-c4c(OC)cccc4)cc3)OCCC2)cc1 | Integrin alpha-4/beta-7 |
| Fc1c(O)ccc(-c2c(N[C@@H](C)C=3N(c4ccccc4)C(=O)c4c(C)ccn4N=3)ncnc2N)c1 | Phosphatidylinositol 3-kinase regulatory subunit alpha/4 5-bisphosphate 3-kinase catalytic subunit alpha isoform |
| Brc1cc(OC(F)(F)F)c(CNC2CCC(Nc3nc(N(C)C)c4c(n3)cccc4)CC2)cc1 | Neuropeptide Y receptor type 5 |
| Brc1cc(OC(F)(F)F)c(C(=O)NCC2CCC(Nc3nc(N(C)C)c4c(n3)cccc4)CC2)cc1 | Neuropeptide Y receptor type 5 |
| S(=O)(=O)(Nc1cc(-c2c3c(N[C@@H](C)C=4N(c5ccccc5)C(=O)c5c(C)ccn5N=4)ncnc3[nH]c2)c2c([nH]cc2)c1)C | Phosphatidylinositol 3-kinase regulatory subunit alpha/4 5-bisphosphate 3-kinase catalytic subunit alpha isoform |
| O=C(O)[C@@H](NC(=O)[C@]1(CCCCN(C)C)OCCC1)Cc1ccc(-c2c(OC)cccc2OC)cc1 | Integrin alpha-4/beta-7 |
| O=C(O)[C@@H](NC(=O)[C@]1(c2oc3c(n2)cccc3)OCCC1)Cc1ccc(-c2c(OC)cccc2OC)cc1 | Integrin alpha-4/beta-7 |
| S(c1c2c(N[C@@H](C)C=3N(c4ccccc4)C(=O)c4c(C)ccn4N=3)ncnc2[nH]c1)c1cc(O)ccc1 | Phosphatidylinositol 3-kinase regulatory subunit alpha/4 5-bisphosphate 3-kinase catalytic subunit beta isoform |
| Brc1ccc([C@@]2(C(=O)N[C@H](C(=O)O)Cc3ccc(-c4c(OC)cccc4)cc3)OCCC2)cc1 | Integrin alpha-4/beta-7 |
| S(c1c2c(N[C@@H](C)C=3N(c4ccccc4)C(=O)c4c(C)ccn4N=3)ncnc2[nH]c1)c1cc(O)ccc1 | Phosphatidylinositol 3-kinase regulatory subunit alpha/4 5-bisphosphate 3-kinase catalytic subunit alpha isoform |
| O=C1N(c2ccccc2)C([C@@H](Nc2ncnc3[nH]cc(Cc4cc(O)ccc4)c23)C)=Nn2c1c(C)cc2 | Phosphatidylinositol 3-kinase regulatory subunit alpha/4 5-bisphosphate 3-kinase catalytic subunit beta isoform |
| O=C(O)[C@@H](NC(=O)[C@]1(c2ccc(C(C)(C)C)cc2)OCCC1)Cc1ccc(-c2c(OC)cccc2)cc1 | Integrin alpha-4/beta-7 |
| O=C1N(c2ccccc2)C([C@@H](Nc2ncnc3[nH]cc(Cc4cc(O)ccc4)c23)C)=Nn2c1c(C)cc2 | Phosphatidylinositol 3-kinase regulatory subunit alpha/4 5-bisphosphate 3-kinase catalytic subunit alpha isoform |

Table S8 Positions, bond types, distances, and energy values of the interaction relationships among top five ranked compounds-protein pairs.

| Rank | Ligand (Position) | Receptor  (Position) | Interaction | Distance (Å) | E (kcal/mol) |
| --- | --- | --- | --- | --- | --- |
| 1 | CL (1) | ASN (16 A) | H-donor | 3.10 | -0.6 |
|  | O (17) | ASN (42 C) | H-acceptor | 3.00 | -0.7 |
|  | O (23) | ASN (51 C) | H-acceptor | 3.10 | -1.3 |
|  | 6-ring | ALA (47 A) | pi-H | 3.69 | -0.8 |
| 2 | C (27) | ARG (1253 A) | H-donor | 3.08 | -0.5 |
|  | N (46) | MET (1199 A) | H-acceptor | 3.46 | -0.8 |
|  | 5-ring | LEU (1122 A) | pi-H | 4.07 | -1.3 |
|  | 6-ring | VAL (1130 A) | pi-H | 4.59 | -0.5 |
|  | 5-ring | GLY (1202 A) | pi-H | 4.07 | -2.7 |
| 3 | CL(1) | SER (48 A) | H-donor | 3.51 | -0.7 |
|  | O (24) | ASP (90 A) | H-donor | 2.79 | -7.9 |
|  | C (36) | GLU (87 A) | H-donor | 3.39 | -0.5 |
|  | 6-ring | ARG (89 A) | pi-cation | 3.88 | -0.8 |
| 4 | C (5) | ASP (125 B) | H-donor | 3.41 | -0.6 |
|  | N (14) | GLY (127 B) | H-donor | 2.87 | -1.5 |
|  | N (64) | ASN (30 A) | H-donor | 3.45 | -0.7 |
|  | O (2) | GLY (49 A) | H-acceptor | 3.07 | -0.5 |
| 5 | O (1) | GLY (28 A) | H-acceptor | 3.59 | -0.7 |
|  | C (55) | TYR (189 A) | H-pi | 4.59 | -0.8 |
|  | 6-ring | VAL (95 A) | pi-H | 4.51 | -0.6 |

Reference:

1. Zheng, Y., P. Tang, W. Qiu, H. Wang, J. Guo, and Z. Huang. *A Novel Deep Learning Framework for Interpretable Drug-Target Interaction Prediction with Attention and Multi-task Mechanism*. in *Database Systems for Advanced Applications*. 2023. Cham: Springer Nature Switzerland.

2. Wang, Z., L. Liang, Z. Yin, and J. Lin, *Improving chemical similarity ensemble approach in target prediction.* Journal of Cheminformatics, 2016. **8**(1): p. 20.

3. Chatterjee, A., R. Walters, Z. Shafi, O.S. Ahmed, M. Sebek, D. Gysi, R. Yu, T. Eliassi-Rad, A.-L. Barabási, and G. Menichetti, *Improving the generalizability of protein-ligand binding predictions with AI-Bind.* Nature Communications, 2023. **14**(1): p. 1989.

4. Lim, J., S. Ryu, K. Park, Y.J. Choe, J. Ham, and W.Y. Kim, *Predicting Drug–Target Interaction Using a Novel Graph Neural Network with 3D Structure-Embedded Graph Representation.* Journal of Chemical Information and Modeling, 2019. **59**(9): p. 3981-3988.

5. Torng, W. and R.B. Altman, *Graph Convolutional Neural Networks for Predicting Drug-Target Interactions.* Journal of Chemical Information and Modeling, 2019. **59**(10): p. 4131-4149.

6. Zeng, X., S. Zhu, Y. Hou, P. Zhang, L. Li, J. Li, L.F. Huang, S.J. Lewis, R. Nussinov, and F. Cheng, *Network-based prediction of drug-target interactions using an arbitrary-order proximity embedded deep forest.* Bioinformatics, 2020. **36**(9): p. 2805-2812.

7. Zeng, X., S. Zhu, W. Lu, Z. Liu, J. Huang, Y. Zhou, J. Fang, Y. Huang, H. Guo, L. Li, B.D. Trapp, R. Nussinov, C. Eng, J. Loscalzo, and F. Cheng, *Target identification among known drugs by deep learning from heterogeneous networks.* Chem Sci, 2020. **11**(7): p. 1775-1797.

8. Rifaioglu, A.S., V. Atalay, M.J. Martin, R. Cetin-Atalay, and Tunca, *DEEPScreen: High Performance Drug-Target Interaction Prediction with Convolutional Neural Networks Using 2-D Structural Compound Representations†.* Chemical ence, 2020.

9. Lee, I., J. Keum, and H. Nam, *DeepConv-DTI: Prediction of drug-target interactions via deep learning with convolution on protein sequences.* PLOS Computational Biology, 2019. **15**(6): p. e1007129.

10. Huang, Y., H.-Y. Huang, Y. Chen, Y.-C.-D. Lin, L. Yao, T. Lin, J. Leng, Y. Chang, Y. Zhang, Z. Zhu, K. Ma, Y.-N. Cheng, T.-Y. Lee, and H.-D. Huang, *A Robust Drug–Target Interaction Prediction Framework with Capsule Network and Transfer Learning.* International Journal of Molecular Sciences, 2023. **24**(18): p. 14061.

11. Gao, M., D. Zhang, Y. Chen, Y. Zhang, Z. Wang, X. Wang, S. Li, Y. Guo, G.I. Webb, A.T.N. Nguyen, L. May, and J. Song, *GraphormerDTI: A graph transformer-based approach for drug-target interaction prediction.* Computers in Biology and Medicine, 2024. **173**: p. 108339.

12. Cer, R.Z., U. Mudunuri, R. Stephens, and F.J. Lebeda, *IC50-to-Ki: a web-based tool for converting IC50 to Ki values for inhibitors of enzyme activity and ligand binding.* Nucleic Acids Res, 2009. **37**(Web Server issue): p. W441-5.
